# Correction: *De Novo* Peptide Design and Experimental Validation of Histone Methyltransferase Inhibitors

**DOI:** 10.1371/journal.pone.0095535

**Published:** 2014-04-16

**Authors:** The PLOS ONE Staff

## Notice of Republication

This article was republished on March 27, 2014, to correct the figure order. Please download the article again to view the correct version. The originally published, uncorrected article and the republished, corrected article are provided here for reference.

## Supporting Information

File S1 Originally published, uncorrected article.
(PDF)

File S2 Republished, corrected article.
(PDF)
