# Supplementary figures and images for: De Novo Peptide Design and Experimental Validation of Histone Methyltransferase Inhibitors
Source: PLoS One. 2014 Feb 28;9(2):e90095. doi: 10.1371/journal.pone.0090095 (PMC3938834; doi:10.1371/journal.pone.0090095)

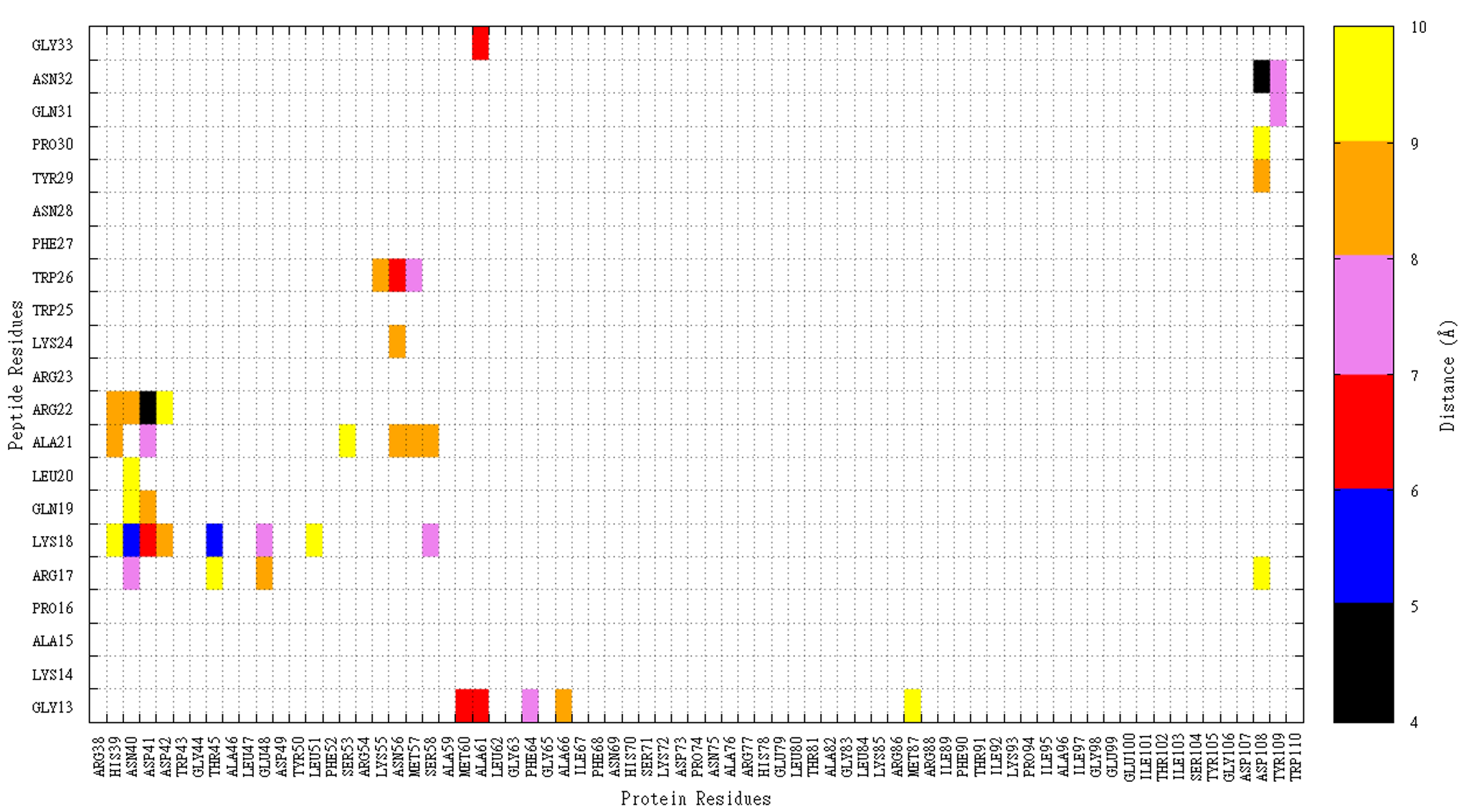

Supplement: Figure S1 — Contact Map for a Top Bound Structure of SQ037, cd2G46_ppk.0383.pdb. Contact map for one of the top bound structures produced for the top designed inhibitor SQ037, cd2G46 ppk.0383.pdb. All protein position numbers correspond to the numbering given in PDB:2G46. All peptide position numbers correspond to the numbering used in Table 1. Distances are given in Å, and only contacts between 4 Å–10 Å are visualized. (TIF) [file pone.0090095.s001.tif]

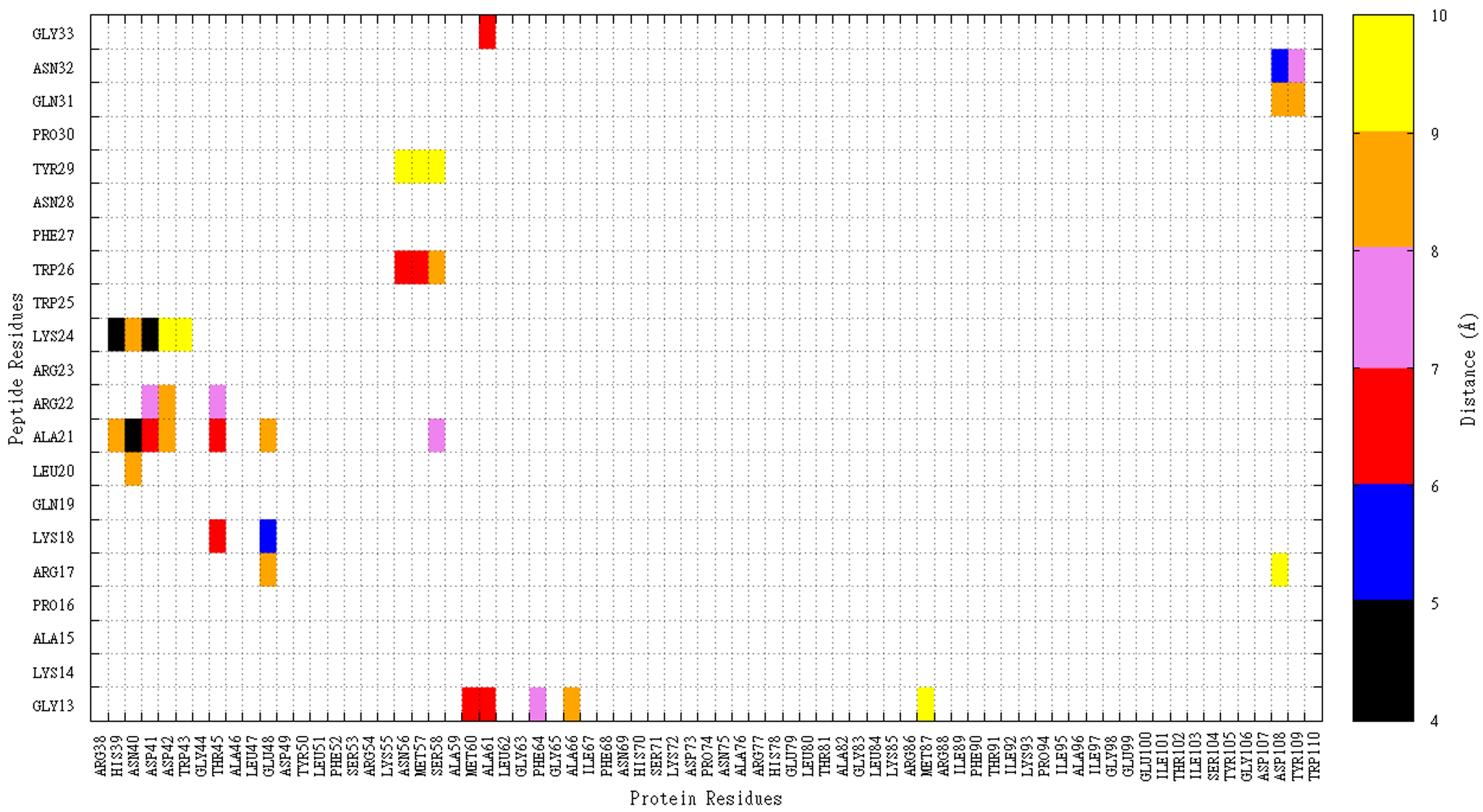

Supplement: Figure S2 — Contact Map for a Top Bound Structure of SQ037, cd2G46_ppk.0514.pdb. Contact map for one of the top bound structures produced for the top designed inhibitor SQ037, cd2G46 ppk.0514.pdb. All protein position numbers correspond to the numbering given in PDB:2G46. All peptide position numbers correspond to the numbering used in Table 1. Distances are given in Å, and only contacts between 4 Å–10 Å are visualized. (TIF) [file pone.0090095.s002.tif]

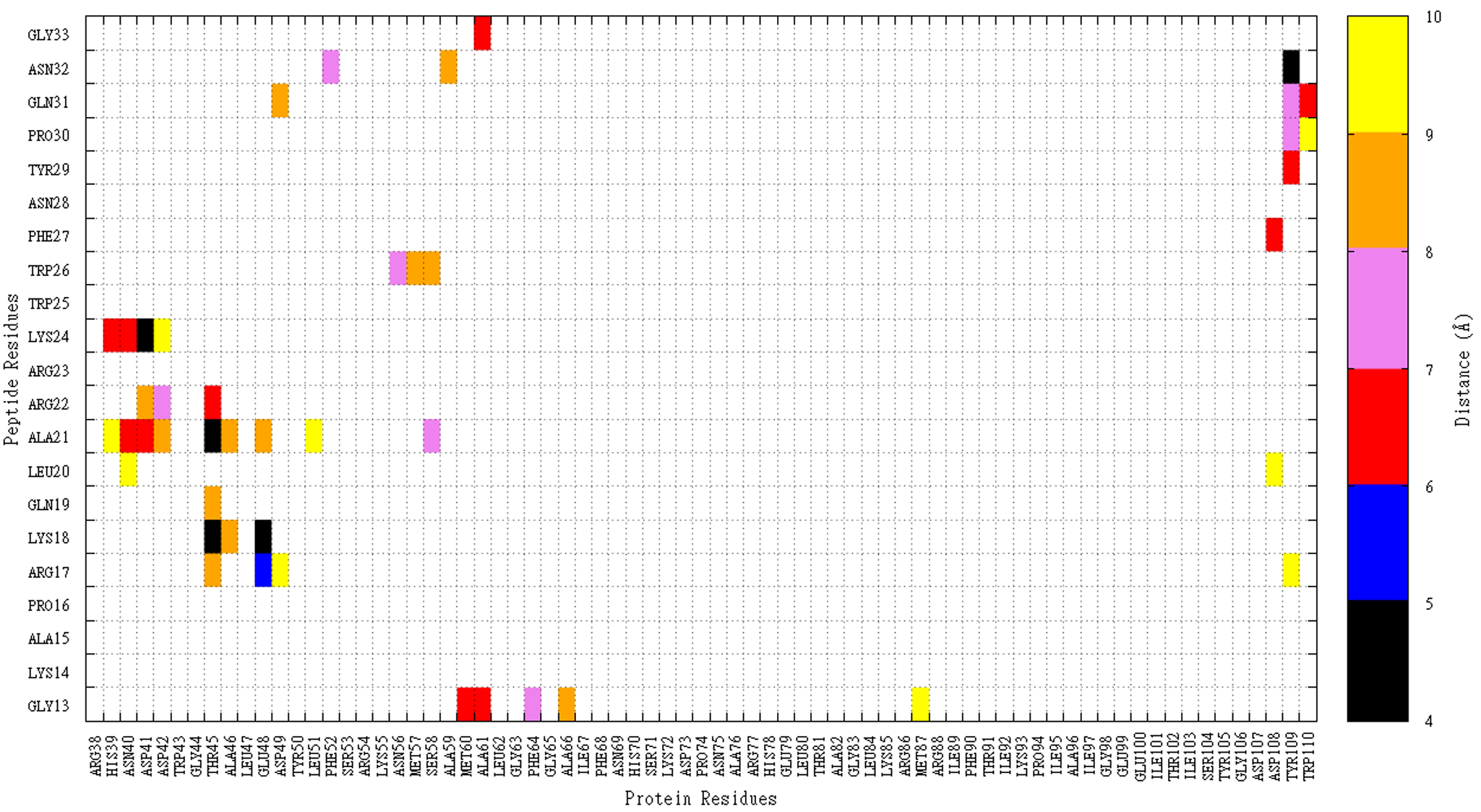

Supplement: Figure S3 — Contact Map for a Top Bound Structure of SQ037, cd2G46_ppk.1010.pdb. Contact map for one of the top bound structures produced for the top designed inhibitor SQ037, cd2G46 ppk.1010.pdb. All protein position numbers correspond to the numbering given in PDB:2G46. All peptide position numbers correspond to the numbering used in Table 1. Distances are given in Å, and only contacts between 4 Å–10 Å are visualized. (TIF) [file pone.0090095.s003.tif]

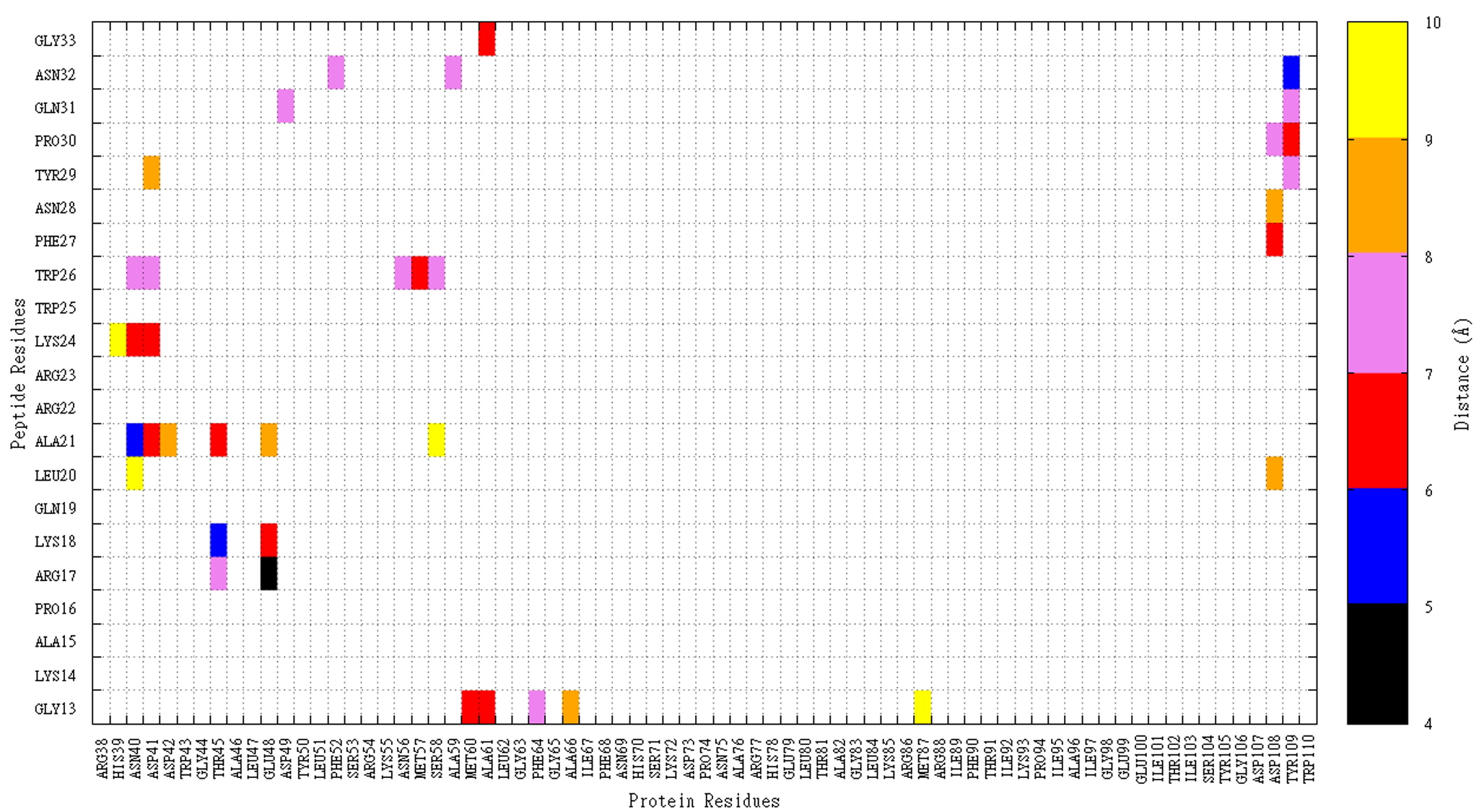

Supplement: Figure S4 — Contact Map for a Top Bound Structure of SQ037, cd2G46_ppk.1330.pdb. Contact map for one of the top bound structures produced for the top designed inhibitor SQ037, cd2G46 ppk.1330.pdb. All protein position numbers correspond to the numbering given in PDB:2G46. All peptide position numbers correspond to the numbering used in Table 1. Distances are given in Å, and only contacts between 4 Å–10 Å are visualized. (TIF) [file pone.0090095.s004.tif]

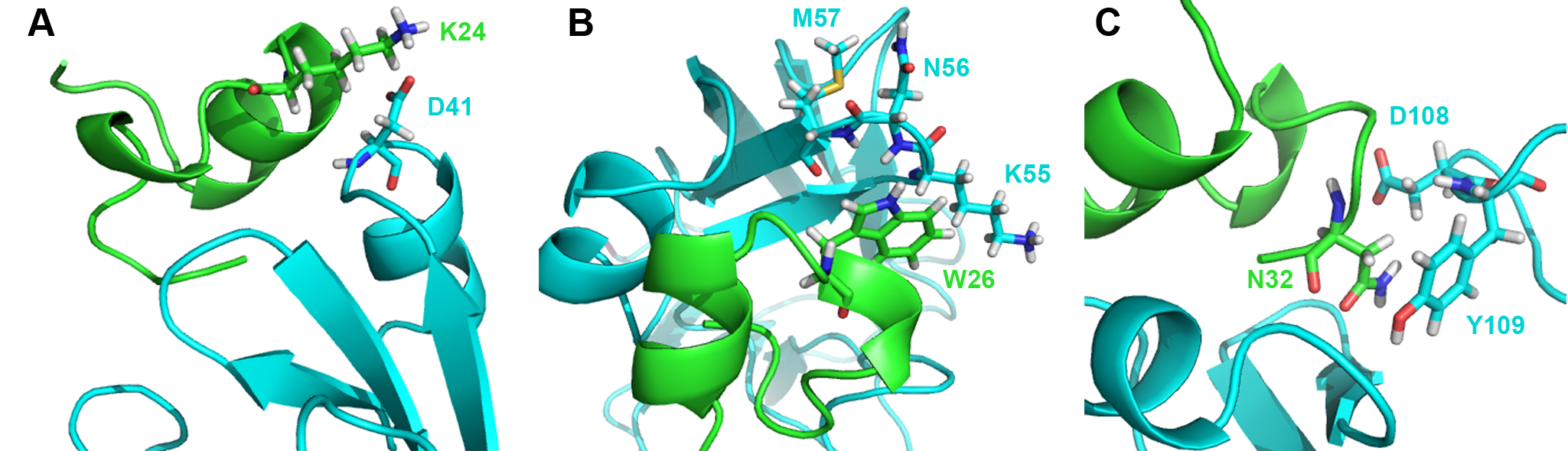

Supplement: Figure S5 — Contact Highlights for the Bound Structure of Sequence SQ037. Low energy structure for SQ037 with several important protein (blue) and peptide (green) positions labelled. Different angles are provided to highlight contacts with peptide positions (A) K24, (B) W26, and (C) N32. (TIF) [file pone.0090095.s005.tif]

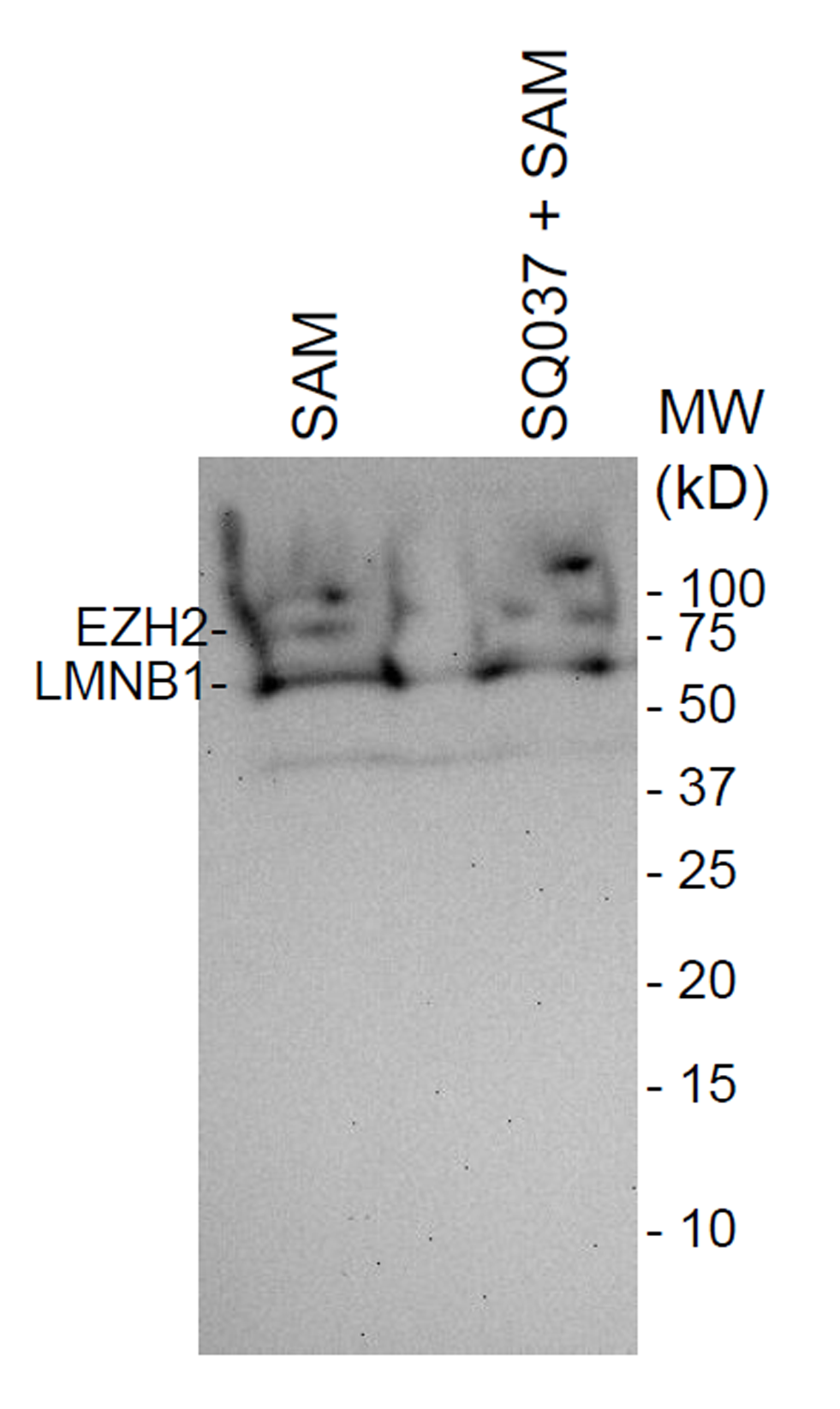

Supplement: Figure S6 — Western Blot EZH2 Degredation Experiments. Western Blot analysis comparing levels of human EZH2 (≈ 98 kD, Cell Signaling) and human lamin B1 (≈ 66 kD, Invitrogen) from extracts of in nucleo reactions containing or lacking the SQ037 inhibitor peptide. (TIF) [file pone.0090095.s006.tif]
