# Supplementary material for: De Novo Peptide Design and Experimental Validation of Histone Methyltransferase Inhibitors
Source: PLoS One. 2014 Feb 28;9(2):e90095. doi: 10.1371/journal.pone.0090095 (PMC3938834; doi:10.1371/journal.pone.0090095)
Supplement: Table S1 — Results for Sequences Tested by Approximate Binding Affinity Validation. Rankings and exact calculated values are given for sequence selection (potential energy rank #1 = lowest potential energy, ), fold specificity (fold specificity rank #1 = highest specificity, ), and approximate binding affinity (approximate binding affinity rank #1 = highest affinity, ). and were not calculated for the native sequence. * indicated peptide tested experimentally. (DOCX) [file pone.0090095.s007.docx]

**Supplementary Table S1: Results for Sequences Tested by Approximate Binding Affinity Validation**

| **Name** | **K* Rank** | **K*** | **FS Rank** | **Fold Spec** | **E Rank** | **Sequence** | **Run #** |
| --- | --- | --- | --- | --- | --- | --- | --- |
| SQ040* | 1 | 6.65E+40 | 2 | 53.984 | 210 | GKAPRKQLANRKWWKNYPRDG | 4 |
| SQ043* | 2 | 3.63E+21 | 3 | 44.521 | 306 | GKAPRKQLARKNWWKNYPRDG | 4 |
| SQ035* | 3 | 2.93E+12 | 6 | 3.432 | 372 | GKAPRKQLATSAAWKPARATK | 3 |
| SQ037* | 4 | 1.80E+11 | 1 | 62.087 | 500 | GKAPRKQLARRKWWFNYPQNG | 4 |
| SQ032* | 5 | 1.17E+11 | 5 | 4.046 | 299 | GKAPRKQLATPAWRASAKATK | 3 |
| SQ020* | 6 | 1.29E+09 | 7 | 2.188 | 438 | GKAPRKQLASKAARAATPKTG | 2 |
| SQ026* | 7 | 3.11E+06 | 4 | 7.318 | 363 | GKAPRKQLAPKAATKNARATS | 3 |
| SQ010* | 8 | 2.69E+03 | 8 | 2.098 | 216 | GKAPRKQLASAKKAAATPRTG | 1 |
| SQ025* | 9 | 1.00E+03 | 10 | 2.028 | 494 | GKAPRKQLAAKAATKSAPRTG | 2 |
| SQ028 | - | 1.42E+02 | - | 4.626 | 442 | GKAPRKQLAPTAAWKSAKATR | 3 |
| SQ011* | 10 | 4.32E+01 | 9 | 2.095 | 428 | GKAPRKQLAAKSAAAKTPRTG | 1 |
| SQ031 | - | 7.18E-01 | - | 4.074 | 226 | GKAPRKQLAPKAARKNATATS | 3 |
| SQ004 | - | 5.33E-01 | - | 2.475 | 317 | GKAPRKQLAASKAAAKTPRTG | 1 |
| SQ003 | - | 5.24E-01 | - | 2.744 | 248 | GKAPRKQLASKAAAAKTPRTG | 1 |
| SQ013 | - | 1.03E-02 | - | 2.016 | 484 | GKAPRKQLASTKKAAATPRAG | 1 |
| SQ023 | - | 5.65E-03 | - | 2.059 | 304 | GKAPRKQLATKAKAASAPRTG | 2 |
| SQ041 | - | 4.60E-03 | - | 51.253 | 16 | GKAPRKQLARNKWWKNYPRDG | 4 |
| Native | - | 2.96E-04 | - | - | - | GKAPRKQLATKAARKSAPATG | 1 |
| SQ005 | - | 7.73E-05 | - | 2.336 | 376 | GKAPRKQLAAAKSAAKTPRTG | 1 |
| SQ036 | - | 3.98E-07 | - | 82.994 | 475 | GKAPRKQLARNNWWKKYPRDG | 4 |
| SQ022 | - | 3.47E-07 | - | 2.067 | 244 | GKAPRKQLAAKAAAKSTPRTG | 2 |
| SQ030 | - | 1.37E-07 | - | 4.506 | 234 | GKAPRKQLAPKAARKNATAST | 3 |
| SQ002 | - | 3.39E-08 | - | 2.855 | 330 | GKAPRKQLASAAKAAKTPRTG | 1 |
| SQ044 | - | 3.07E-08 | - | 43.158 | 407 | GKAPRKQLARKNWWKNLPRDG | 4 |
| SQ042 | - | 1.52E-09 | - | 50.816 | 20 | GKAPRKQLARNKWWKNLPRDG | 4 |
| SQ016 | - | 1.52E-09 | - | 2.662 | 272 | GKAPRKQLASKAAAKATPRTG | 2 |
| SQ009 | - | 6.82E-10 | - | 2.106 | 106 | GKAPRKQLAAKASAAKTPRTG | 1 |
| SQ021 | - | 7.65E-11 | - | 2.115 | 393 | GKAPRKQLAKAAAAKSTPRTG | 2 |
| SQ018 | - | 6.95E-11 | - | 2.3 | 400 | GKAPRKQLATKTARASAPAKG | 2 |
| SQ014 | - | 5.52E-11 | - | 2.854 | 454 | GKAPRKQLATKASAAKAPRTG | 2 |
| SQ033 | - | 3.65E-11 | - | 3.554 | 163 | GKAPRKQLAPKAAWKTASATR | 3 |
| SQ017 | - | 1.38E-11 | - | 2.386 | 467 | GKAPRKQLATKSARAATPAKG | 2 |
| SQ034 | - | 1.01E-11 | - | 3.506 | 200 | GKAPRKQLAPKAAAKSWTATR | 3 |
| SQ015 | - | 3.32E-13 | - | 2.766 | 459 | GKAPRKQLASKAATAKAPRTG | 2 |
| SQ027 | - | 7.80E-14 | - | 4.722 | 273 | GKAPRKQLAPKAARKNASATT | 3 |
| SQ007 | - | 6.30E-14 | - | 2.226 | 304 | GKAPRKQLAKSAAAAKTPRTG | 1 |
| SQ001 | - | 8.00E-15 | - | 5.845 | 370 | GKAPRKQLAAKRKAATAPSTG | 1 |
| SQ019 | - | 0.00E+00 | - | 2.232 | 375 | GKAPRKQLASKTARATAPAKG | 2 |
| SQ012 | - | 0.00E+00 | - | 2.041 | 439 | GKAPRKQLASTKKAAATPARG | 1 |
| SQ024 | - | 0.00E+00 | - | 2.046 | 419 | GKAPRKQLATKTKAASAPARG | 2 |
| SQ029 | - | 0.00E+00 | - | 4.521 | 302 | GKAPRKQLAPTAAWKSARATK | 3 |
| SQ008 | - | 0.00E+00 | - | 2.182 | 264 | GKAPRKQLAASAKAAKTPRTG | 1 |
| SQ045 | - | 0.00E+00 | - | 39.308 | 453 | GKAPRKQLANNKWWFKYPQRG | 4 |
| SQ006 | - | 0.00E+00 | - | 2.238 | 265 | GKAPRKQLAKAASAAKTPRTG | 1 |
| SQ039 | - | 0.00E+00 | - | 55.398 | 270 | GKAPRKQLANRKWWKNLPRDG | 4 |
| SQ038 | - | 0.00E+00 | - | 56.421 | 398 | GKAPRKQLARNKWWKNYPDRG | 4 |

Rankings and exact calculated values are given for sequence selection (lowest energy = 1, E), fold specificity (highest specificity = 1, fspec), and approximate binding affinity (highest affinity = 1, K ∗). E and fspec were not calculated for the native sequence. * indicated peptide tested experimentally.
