# Supplementary material for: De Novo Peptide Design and Experimental Validation of Histone Methyltransferase Inhibitors
Source: PLoS One. 2014 Feb 28;9(2):e90095. doi: 10.1371/journal.pone.0090095 (PMC3938834; doi:10.1371/journal.pone.0090095)
Supplement: Table S2 — Relative Abundance of All Peptides Corresponding to a Given Methylated State. Relative abundance of all peptides corresponding to a given methylated state containing at least one unlabeled 12CH3-methyl group from in nucleo reactions performed with 100 M control or SQ037 peptide. Thus, for H3K27me3, the relative abundance corresponds to (H3K27me3:0+H3K27me3:1+H3K27me3:2)/(H3K27me3:0+H3K27me3:1+H3K27me3:2+H3K27me3:3). H3K9me1 corresponds to the monomethylated 9–17 H3 peptide (KSTGGKAPR), H4K20me1 and me2 correspond to the 20–23 H4 peptide (KVLR) monomethylated and dimethylated on K20 respectively, H3K36me1 and H3K36me2 correspond to the 27–40 H3 peptide (KSAPATGGVKKPHR) monomethylated and dimethylated on K36 respectively, and H3K79me1 and H3K79me2 correspond to the 73–83 H3 peptide (EIAQDFKTDLR) monomethylated and dimethylated on K79 respectively. (DOCX) [file pone.0090095.s008.docx]

**Supplementary Table S2**: **Relative Abundance of All Peptides Corresponding to a Given Methylated State**

|  | H3K27me3 | H3K9me1 | H4K20me1 | H4K20me2 | H3K36me1 | H3K36me2 | H3K79me1 | H3K79me2 |
| --- | --- | --- | --- | --- | --- | --- | --- | --- |
| Control | 0.2270 | 0.0614 | 0.0639 | 0.1316 | 0.0612 | 0.1208 | 0.0807 | 0.1713 |
| SQ037 | 0.1903 | 0.0571 | 0.0644 | 0.1309 | 0.0676 | 0.1129 | 0.0763 | 0.1756 |
| Difference | 0.0367 | 0.0043 | -0.0005 | 0.0007 | -0.0064 | 0.0079 | 0.0044 | -0.0043 |

Relative abundance of all peptides corresponding to a given methylated state containing at least one unlabeled ^12^CH_3_-methyl group from *in nucleo* reactions performed with 100 µM control or SQ037 peptide. Thus, for H3K27me3, the relative abundance corresponds to (H3K27me3:0+H3K27me3:1+H3K27me3:2)/(H3K27me3:0+H3K27me3:1+H3K27me3:2+H3K27me3:3). H3K9me1 corresponds to the monomethylated 9-17 H3 peptide (KSTGGKAPR), H4K20me1 and me2 correspond to the 20-23 H4 peptide (KVLR) monomethylated and dimethylated on K20 respectively, H3K36me1 and H3K36me2 correspond to the 27-40 H3 peptide (KSAPATGGVKKPHR) monomethylated and dimethylated on K36 respectively, and H3K79me1 and H3K79me2 correspond to the 73-83 H3 peptide (EIAQDFKTDLR) monomethylated and dimethylated on K79 respectively.
