# Supplementary material for: Correction: De Novo Peptide Design and Experimental Validation of Histone Methyltransferase Inhibitors
Source: PLoS One. 2014 Apr 16;9(4):e95535. doi: 10.1371/journal.pone.0095535 (PMC3989331; doi:10.1371/journal.pone.0095535)
Supplement: File S1 — Originally published, uncorrected article. (PDF) [file pone.0095535.s001.pdf]

De Novo Peptide Design and Experimental Validation of Histone Methyltransferase Inhibitors

James Smadbeck[1], Meghan B. Peterson[1], Barry M. Zee[2], Shivani Garapaty[3], Aashna Mago[2], Christina Lee[3], Athanassios Giannis[4], Patrick Trojer[3], Benjamin A. Garcia[2,5,6], Christodoulos A. Floudas[1]*

1 Department of Chemical and Biological Engineering, Princeton University, Princeton, New Jersey, United States of America, **2** Department of Molecular Biology, Princeton University, Princeton, New Jersey, United States of America, **3** Constellation Pharmaceuticals, Cambridge, Massachusetts, United States of America, **4** University of Leipzig, Institute of Organic Chemistry, Leipzig, Germany, **5** Department of Chemistry, Princeton University, Princeton, New Jersey, United States of America, **6** Epigenetics Program, Department of Biochemistry and Biophysics, Perelman School of Medicine, University of Pennsylvania, Philadelphia, Pennsylvania, United States of America

Abstract

Histones are small proteins critical to the efficient packaging of DNA in the nucleus. DNA–protein complexes, known as nucleosomes, are formed when the DNA winds itself around the surface of the histones. The methylation of histone residues by enhancer of zeste homolog 2 (EZH2) maintains gene repression over successive cell generations. Overexpression of EZH2 can silence important tumor suppressor genes leading to increased invasiveness of many types of cancers. This makes the inhibition of EZH2 an important target in the development of cancer therapeutics. We employed a three-stage computational *de novo* peptide design method to design inhibitory peptides of EZH2. The method consists of a sequence selection stage and two validation stages for fold specificity and approximate binding affinity. The sequence selection stage consists of an integer linear optimization model that was solved to produce a rank-ordered list of amino acid sequences with increased stability in the bound peptide-EZH2 structure. These sequences were validated through the calculation of the fold specificity and approximate binding affinity of the designed peptides. Here we report the discovery of novel EZH2 inhibitory peptides using the *de novo* peptide design method. The computationally discovered peptides were experimentally validated *in vitro* using dose titrations and mechanism of action enzymatic assays. The peptide with the highest *in vitro* response, SQ037, was validated *in nucleo* using quantitative mass spectrometry-based proteomics. This peptide had an IC_{50} of 13.5 μM, demonstrated greater potency as an inhibitor when compared to the native and K27A mutant control peptides, and demonstrated competitive inhibition versus the peptide substrate. Additionally, this peptide demonstrated high specificity to the EZH2 target in comparison to other histone methyltransferases. The validated peptides are the first computationally designed peptides that directly inhibit EZH2. These inhibitors should prove useful for further chromatin biology investigations.

Citation: Smadbeck J, Peterson MB, Zee BM, Garapaty S, Mago A, et al. (2014) *De Novo* Peptide Design and Experimental Validation of Histone Methyltransferase Inhibitors. PLoS ONE 9(2): e90095. doi:10.1371/journal.pone.0090095

Editor: H. Sunny Sun, National Cheng Kung University Medical College, Taiwan

Received July 29, 2013; **Accepted** January 30, 2014; **Published** February 28, 2014

Copyright: © 2014 Smadbeck et al. This is an open-access article distributed under the terms of the Creative Commons Attribution License, which permits unrestricted use, distribution, and reproduction in any medium, provided the original author and source are credited.

Funding: CAF acknowledges support from the National Institutes of Health (NIH; 5R01 GM052032). BAG and CAF acknowledge support from the National Science Foundation (NSF; CBET-091143). BAG also acknowledges support from an NSF Early Faculty CAREER award, and the NIH Innovator award (DP2OD007447) from the Office of the Director, NIH. BMZ is supported by an NSF graduate research fellowship. The funders had no role in study design, data collection and analysis, decision to publish, or preparation of the manuscript.

Competing Interests: The authors would like to explicitly declare the affiliation of Dr. Shivani Garapaty, Christina Lee, and Dr. Patrick Trojer as employees of Constellation Pharmaceuticals at the time this study was performed. Their affiliation with Constellation Pharmaceuticals does not alter the authors' adherence to all the PLOS ONE policies on sharing data and materials.

* E-mail: floudas@titan.princeton.edu

Introduction

Histones are small proteins critical to the efficient packaging of DNA in the nucleus [1]. DNA winds itself around the surface of the histones, forming DNA-protein complexes known as nucleosomes [2]. The N-terminal histone tail protrudes from the nucleosome, allowing for post-translational modification of key histone residues. These post-translational modifications commonly consist of phosphorylation, acetylation, methylation, ubiquitylation, ribosylation, and sumoylation, to name a few [3]. Combinations of such histone modifications take part in the regulation of DNA transcription and constitute an additional level to the genetic code, termed the "histone code". These modifications are dynamically maintained by various histone-modifying enzymes that control their transfer and removal.

While histone-modifying enzymes are important for normal cell function, overexpression of the enzymes can result in the aberrant silencing of genes that are required to govern cell identity. For example, enhancer of zeste homolog 2 (EZH2) is a SET-domain containing histone methyltransferase (HMT) that catalyzes the di- and trimethylation of the lysine at position 27 of histone H3 (H3K27) [4]. Both methylation states of H3K27 have been linked to heterochromatic genomic regions and to epigenetic silencing [4]. Overabundance of EZH2 has been linked to the silencing of more than 100 genes in prostate cell lines, including several important tumor suppressors [5]. For this reason, the overexpression of EZH2 has been correlated to the invasiveness of breast and prostate cancer [6,7] and linked to various other cancer types [8]. Moreover, recurrent mutations of EZH2 have been found in

germinal center B-cell like diffuse large B-cell lymphoma, follicular lymphoma, and melanoma [9]. The mutated residues alter the substrate specificity of EZH2 and facilitate the conversion from a dimethylated to a trimethylated state, thus resulting in significantly elevated global H3K27me3 levels. Cancer cells harboring EZH2 mutations were recently shown to be dependent on the EZH2 catalytic activity since their viability was severely affected by EZH2 small molecule inhibitors [9]. Additionally, studies have shown that RNAi mediated knockdown of EZH2 inhibits the growth and migration of cancer cells and upregulates the tumor suppressor gene BRCA1 [10]. This makes the inhibition of histone-modifying enzymes, in particular EZH2, an important target in the development of cancer therapeutics for many different cancer types.

Histone methyltransferase small molecule inhibitors obtained through random, large-scale screening of compound libraries have been reported in the literature [4,11–17]. However, the number of potent and selective inhibitors remains small and the community still does not have adequate tools to target all methyltransferases that are implicated in human disease. For this reason EZH2 remains an important target for inhibitor design. The pharmacological properties of peptidic inhibitors make their use in the development of cancer therapeutics difficult. However, the specificity with which they can act with their binding partner make them desirable for the development of chemical probes for the interrogation of methyltransferase and chromatin biology [18]. Peptide inhibitors are generally more specific than small molecule inhibitors as they often more closely resemble the natural binders of many target proteins.

The aim of this work was to find specific peptidic inhibitors of EZH2 using a computational *de novo* peptide design framework. This framework consists of three stages. The first stage is an optimization-based sequence selection stage that aims for stability of the designed sequence in the given peptide template structure through the minimization of a potential energy. The second stage determines the fold specificity of the peptide for the template structure in comparison to the native structure. The third stage determines the approximate binding affinity of the design peptides for EZH2 in order to assess their preference for the bound versus unbound state. Through these three stages of increasing computational complexity, one aims to produce peptides that are specific for the target EZH2 structure. In addition to the application of the designed peptides as chemical probes for the interrogation of chromatin biology, experimentally validated peptides are of significant importance to the further development of the peptide design framework. Retrospective analysis of the structural template and biological constraints used as input into the sequence selection stage can demonstrate what types of constraints are useful for future methyltransferase design, as well as peptidic inhibitor design as a whole.

Methods

De Novo Peptide Design

The computational, three-stage *de novo* peptide design method used in this study was focused on the development of novel peptidic inhibitors of enhancer of zeste homolog 2 (EZH2) [19–26]. The first stage of the method is a sequence selection stage that uses biologically relevant constraints in an integer linear optimization model to produce a rank ordered list of sequences with the lowest potential energy in a given template structure. The second stage takes the top sequences from the sequence selection stage and determines the specificity that the candidate sequences have for the target peptide template structure. The sequences with the top fold specificity values are then run through a computationally rigorous third stage to calculate the approximate binding affinity of the sequences to the target protein. Those peptides with the highest predicted binding affinity to the target protein are then validated experimentally. Through the stages of this general methodology, the sequence complexity of the problem is reduced in tandem with increased computational complexity. This results in a small number of candidate peptides for experimental validation. The full framework of the method is shown in Figure 1. The computational details of each stage are described in subsequent sections.

Stage I: Sequence Selection

Template Determination. EZH2 is a SET domain-containing methyltransferase that catalyzes the di- and trimethylation of the lysine in position 27 of histone H3 (H3K27) [4]. The methyltransferase is a catalytic subunit of a larger complex called the polycomb repressive complex 2 (PRC2). Besides EZH2, several non-catalytic subunits of the complex are necessary for correct catalytic function. The SET domain has an unusual "thread-the-needle" structure, called a pseudoknot. While the substrate and cofactor bind on opposite ends of the domain, their binding pockets are connected by an inner chamber where the methyl transfer occurs [8].

There are currently no crystal or NMR structures available for the human EZH2 protein. For this reason, a template structure had to be produced either through computational structure prediction or by selecting a template structure with similar function and binding pocket. A set of high quality NMR structures determined for a viral SET domain (vSET) encoded by Paramecium bursaria chlorella virus 1 (PDB code: 2G46) [27] was available with a relevant bound ligand (H3 tail fragment). This template had a sequence identity of 31% with significant conservation in the regions surrounding the binding site. This level of sequence identity is just above the commonly cited threshold for successful homology modeling (30%), and while there were structures with slightly higher sequence identity, none of those structures methylated H3K27. Since both human EZH2 and the vSET domain catalyze the methylation of H3K27, the experimental virus model was chosen as the template for EZH2 inhibitor design. This was done rather than risk disrupting the specific interactions observed in the experimental model through homology modeling and computational redocking of the histone tail fragment. The template has an SAH cofactor and a truncated, 21-amino-acid H3K27 peptide bound to the vSET domain structure. Since the PDB file contains a dimer of the SET domain template, only the monomeric set of chains A and C were used in the design. This template is shown in Figure 2.

Mutation Set, Biological Constraints, and Force Field. The PDB structure of the vSET domain represents a full template for the design of peptidic inhibitors of EZH2. The native binding peptide is a 21-residue histone fragment. The first nine residues of the fragment do not make contact with the SET domain and were not considered in the design (the residues remain fixed). The mutation constraints of the remaining 12 residues (positions 22–33 in the PDB) were determined by calculating the relative side-chain solvent accessible surface area (SASA) for each amino acid position using naccess [28]. Any position with lower than 20% SASA were considered hydrophobic and any position with higher than 50% were considered hydrophilic. Based on the SASA calculations, positions 22, 23, 24, 31, 32, and 33 were allowed to mutate to hydrophilic amino acids (G, N, Q, H, K, R, D, E, S, T, P, A), positions 26, 27, and 29 were allowed to mutate to hydrophobic amino acids (V, I L, M, F, Y, W, T, A), and

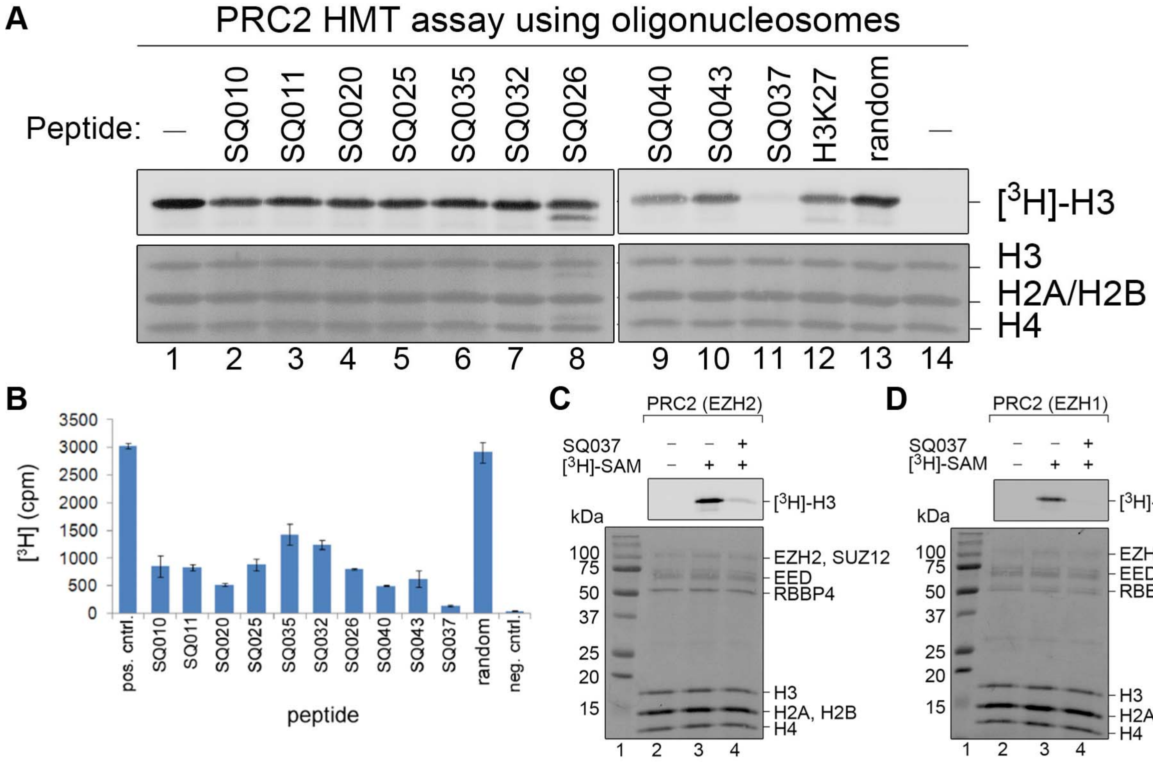

Figure 1. Three-Stage *De Novo* Peptide Design Workflow Diagram. Stage I is an optimization-based sequence selection stage. Stage II is a fold specificity calculation to determine how well designed sequences fold into the desired template structure compared to the native sequence. Stage III is an approximate binding affinity calculation to determine how well the designed sequences binds to the target protein.
doi:10.1371/journal.pone.0090095.g001

positions 25, 28, and 30 were allowed to mutate to all amino acids other than cysteine. The use of hydrophobicity to determine mutation constraints could potential disrupt structure-specific interactions, such as buried salt bridges. For this reason, the native residue was retained as a possible mutation in all positions. This allows the model to retain any specific interactions that are important in binding.

Four separate runs were performed using different, rationally determined biological constraints. For all four runs the mutated positions (22–33 in the PDB) had to maintain their overall native charge of +3. For all but Run 3, position 30 was fixed to proline and position 33 was fixed to glycine, due to the unique structural properties of those amino acids. In addition to these general constraints it was observed that a multiple sequence alignment to relevant peptide sequences showed a complete conservation in the number and type of particular amino acid residues. This is due to the intense evolutionary pressure against the mutation of histone residues. As a result the H3 tail sequences used to generate the constraints are conserved across many organisms. For this reason, the frequency bounds generated for the model allowed only for rearrangements of the sequence. This rearrangement constraint was imposed for Run 1 and Run 2 of the design method. This constraint was relaxed for Run 3 and Run 4. Run 1 allowed only rearrangement of native amino acids (no new amino acids

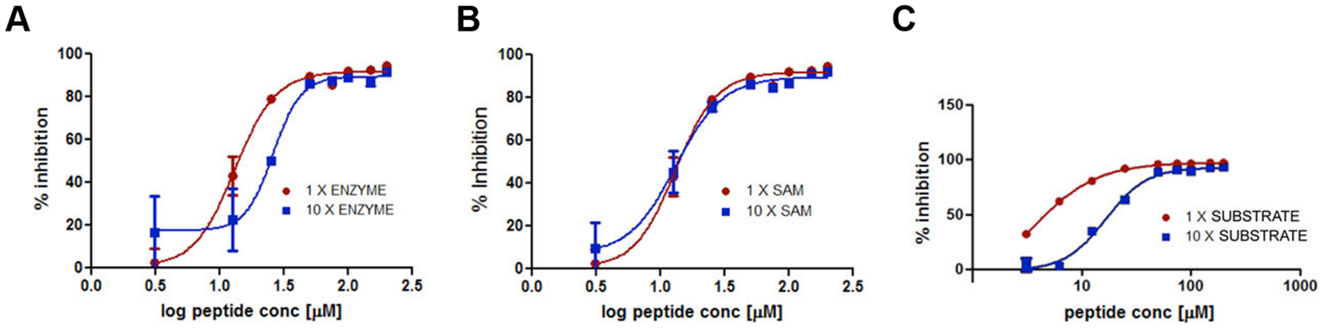

Figure 2. Selected SET Domain Template Structure. (A) Composition of PRC2 showing the complex of SUZ12, EZH2 (containing the SET domain), EED and the association of PHF1. (B) Design template of a histone fragment bound to vSET (grey), PDB code: 2G46. The first nine residues of the histone fragment, which make no contacts with vSET, are colored yellow, while the 21 residues that do make contacts are colored red. The SAH cofactor is colored blue.
doi:10.1371/journal.pone.0090095.g002

allowed). Run 2 was the same as Run 1, but up to only five rearrangements were allowed. Run 3 limited the number of mutations to five, but relaxed the constraint on positions 30 and 33. Finally, Run 4 had no restriction on the number of mutations, but limited the number of each type of amino acid to two. This resulted in an overall computational complexity of approximately 1.8×10^{13} considered peptide sequences of the most relaxed run, Run 4. Besides limiting sequence space, these constraints are meant to provide guidance to the model in producing biologically relevant designed sequences. For example, by observing and mimicking the amino acid frequencies in a set of peptides of similar function, one implicitly accounts for the effect of amino acid content on important biological properties, such as solubility. Peptides that that have amino acid frequencies that fall outside the bounds observed in nature may not have properties suitable for their use in the desired biological setting. Additionally, if there is a consistent charge observed across functionally similar peptides, the charge is assumed to be biologically important and is not allowed to change. This was the case in the methyltransferase inhibitor design, but modifying net charge may be suitable in other applications where a range of charges are observed across similar sequences. In such cases more relaxed bounds on the allowed overall charge of the peptide may be important, not only for the introduction of potentially beneficial salt bridges, but in producing peptides with a wider range of biological properties. In all runs the potential energy force field employed was the 8-bin Centroid-Centroid force field [29]. The force field is a distance bin, binary interaction potential energy force field.

Sequence Selection Optimization Model. The vSET domain design template is flexible, so the distance bin sequence selection method was used to take into account this flexibility. The sequence selection model is a potential energy minimization model as follows [22]:

$$\min_{y_i^j, y_k^l} \sum_{i=1}^{n-1} \sum_{j=1}^{m_i} \sum_{k=i+1}^{n} \sum_{l=1}^{m_k} \sum_{d:disbin_{ikd}=1} E_{ikd}^{jl} w_{ik}^{jl} b_{ikd}$$

subject to

$$\sum_{j=1}^{m_i} y_i^j = 1 \quad \forall \quad i$$

$$\sum_{j=1}^{m_i} w_{ik}^{jl} = y_k^l \quad \forall \quad i,k>i,l$$

$$\sum_{l=1}^{m_k} w_{ik}^{jl} = y_i^j \quad \forall \quad i,k>i,j$$

$$\sum_{d:disbin_{ikd}=1} b_{ikd} = 1 \quad \forall \quad i,k>i$$

$$y_i^j, y_k^l, w_{ik}^{jl}, b_{ikd} = \{0,1\} \quad \forall \quad i,j,k>i,l,d \qquad (1)$$

The model minimizes the summation of pairwise interaction energies E_{ikd}^{jl}, which is the interaction between residue types j and l in residue positions i and k separated by a distance that falls into distance bin d. The binary variable y_i^j equals 1 if residue type j in residue position i, and 0 otherwise. The binary variable w_{ik}^{jl} equals 1 if and only if y_i^j and y_k^l are both equal to 1, and 0 otherwise. This represents an exact linearization of the problem. The final binary variable b_{ikd} is allowed to equal 1 if and only if the distance between positions i and k fall into distance bin d in at least one of the flexible models in the template ($distbin_{ikd} = 1$). This way the model is allowed to select one, and only one, distance bin for two residues to fall into, from those distance bins observed in the flexible template. It is important to understand that the objective function is a minimization of a pairwise interaction potential energy, which takes into account possible structural flexibility and mutational limits through a series of linear constraints. The objective function represents the minimization of free energy in the sequence space and hence this model aims at stability. This model was run using the vSET domain template and four different sets of constraints as described above. For each set of biological constraints, a rank-ordered list of the 500 lowest energy sequences was produced and validated by fold specificity calculation. It should be noted that while the sets of biological constraints were not mutually exclusive (all sequences made in Run 2 fit the constraints of Run 1), no repeated sequences were identified across sequence selection runs.

Stage II: Fold Specificity

The second stage of the computational method assesses how well the designed peptides fold into the given template structure by efficiently calculating the fold specificity of each sequence for the target peptide structure [19,20,23]. Upper and lower bounds for the distances between all Cα atoms, as well as ϕ and ψ dihedral angles of each residue, were calculated from the maximum and minimum values observed across all template models. Using these upper and lower bounds, 500 random conformers were produced using the CYANA 2.1 software package for nuclear magnetic resonance (NMR) structure refinement [30,31]. CYANA performs annealing calculations that simulate a rapid heating of the protein, followed by a slow cooling. Torsion angle dynamics were employed to minimize violations in van der Waals radii bounds and bounds given as part of the flexible template. For each of the 500 structures produced, a local energy minimization was performed using the TINKER 3.6 package [32].

The flexible template ensembles were generated for the native sequence and for each of the four sets of 500 sequences from the sequence selection stage. The specificity of a novel sequence was calculated relative to the native sequence as shown in Equation 2.

$$F_{Spec} = \frac{\sum_{i \epsilon Novel} e^{-\beta E_i}}{\sum_{i \epsilon Native} e^{-\beta E_i}} \qquad (2)$$

Here E_i is the AMBER [33] energy of a given structure i. The parameter β is the thermodynamic beta, where $\beta = \frac{1}{k_B T}$. The sets of native and novel conformers considered were filtered to include only structures that were below upper bounds on the energy and RMSD to the template structure. The bounds were calculated by determining the mean and standard deviation of both RMSD and AMBER energies for the native sequence template set. The upper bound on RMSD was one and a half standard deviations above the mean, while the upper bound on energy was two and a half standard deviations above the mean. These calculations were

performed for the top 500 sequences produced by the sequence selection stage for each of the four runs. In each case, only the portion of the peptide that was allowed to mutate (pos. 22–33 in PDB) was considered in the fold specificity calculation.

Stage III: Approximate Binding Affinity Calculation

All sequences with greater than 2.0 fold specificity for Run 1 and Run 2, along with the top ten sequences from Run 3 and Run 4, were run through a rigorous approximate binding affinity calculation method. This resulted in 45 novel sequences, plus the native sequence, that are provided in Table S1. Lilien et al. [34] proposed an approach for approximate binding affinity calculation based on the generation of ensembles of peptide and ligand structure. The approximate binding affinity of a protein, P, and a peptide, L, is defined as follows:

$$K^* = \frac{q_{PL}}{q_P q_L} \tag{3}$$

where q_{PL}, q_P, q_L are the rotameric conformation ensembles of the complex, free proteins, and free peptide, respectively. These are calculated as follows:

$$q_{PL} = \sum_{b \epsilon B} e^{-\frac{E_b}{RT}}, q_P = \sum_{f \epsilon F} e^{-\frac{E_f}{RT}}, q_L = \sum_{l \epsilon L} e^{-\frac{E_l}{RT}} \tag{4}$$

Where E_b, E_f, and E_l are the Rosetta energy value for a given complex, protein, and peptide conformation, respectively. In this case, 298 K was used for the value of T. To calculate the approximate binding affinity of each novel peptide, the predicted sequences were first run through Rosetta Abinitio [35–37] to calculate a large ensemble of potential peptide structures. A total of 2000 peptide structures were generated for each of the candidate sequences and then clustered by their ϕ and ψ dihedral angles using the OREO clustering method [38,39]. The medoids from the top ten largest conformational clusters, along with the lowest energy structure, were taken as a set of representative structures for each candidate sequence. These representative peptide structures were then docked to the vSET domain structure using RosettaDock [40–42]. Each docking run produced 2000 docked structures, whereby the ten lowest energy structures for each run were taken as representative complex structures (110 total). The representative complex, peptide, and protein structures were then taken and a large ensemble of rotamerically based conformation ensembles were generated using Rosetta Design [43] (22000 complex structures, 22000 peptide structures, and 2000 protein structures). Misdocked peptides were filtered before energetic analysis. The filtered, large conformational ensembles were then used in the approximate binding affinity calculation for each of the 45 candidate peptides. Final results for the candidate peptides are provided in Table 1.

Histone Methyltransferase and Mechanism of Action Enzymatic Assays

The top ten sequences from the approximate binding affinity stage were synthesized by GenScript USA Inc. (Piscataway, NJ) in quantities of 1–4 mg and >95% purity. Experiments were performed using radiometric EZH2 enzymatic assays prepared individually or in a semi-automated fashion on a 384-well platform. For the experiments shown in Figures 3.A, 3.C, and 3.D, 4 μg of oligonucleosomes was used per reaction. For the experiments shown in Figures 3.C–D, 3 μg of PRC2 complex were added. For all other HMT assays (Figure 4, Figure 5, and Table 2) the following balanced conditions (at K_m for cofactor and substrate) were used. Each reaction contained 50 mM Tris (pH 8.5), 3 mM DTT, 0.24 μM ^{3}H-labeled SAM (cofactor, Perkin-Elmer), 10 nM reconstituted recombinant PRC2 (containing full length EZH2, SUZ12, EED, RBAP46/48), 200 nM oligonucleosomes, 6 μM biotinylated H3K27me3 peptides, 0.01% BSA (BSA was omitted in the experiments shown in Figures 3.C–D) and 69 μM BRIJ-35. The putative peptide inhibitors were tested in single point assays and in 10-point dose titrations. The total volume of the reaction mixture was 30 μL. Reactions were incubated for 1 hour at 30°C. Reactions terminated by the addition of 10 μL, 5× Laemmli Sample Buffer were loaded onto 15% Tri-Glycine-Polyacrylamide gels and resolved by PAGE. Resolved proteins were transferred to PVDF membrane at 65 V for 2 hours. Membranes were stained with CBB, destained, dried, and exposed to imaging plates for 24 hours. Images were detected using an FLA-7000 bioimaging system (Fujifilm). Reactions terminated by the addition of SAH to a final concentration of 850 μM were transferred to a 384-well Flashplate (Perkin Elmer) and incubated for 90 min. Liquids were aspirated and wells washed with 1×60 μL of wash buffer (50 mM Tris, pH 8.5, 200 mM NaCl, 0.01% NP-40). Detection of incorporated radioactivity in Flashplates was carried out using a Topcount instrument (Perkin Elmer). Potential inhibitory peptides were tested in single point assays and in 10-point dose titrations. Mechanism of Action Enzymatic Assays were performed under the same conditions and using the same protocol. However, in the Mechanism of Action Enzymatic assays the assays were performed with varied enzyme, substrate, or cofactor concentration to identify the mechanism of inhibition.

In nucleo Methyltransferase Inhibition Reactions

HeLa S3 cells were grown in Joklik modified Dulbecco€s Modified Eagle Medium supplemented with 10% newborn calf serum (Hyclone), 1% Glutamax (Invitrogen), and 1% penicillin/streptomycin antibiotics at a cellular density of approximately $5x10^5$ cells/ml. Additionally, this media lacked $^{12}CH_3$-methionine, but was instead supplemented with $^{13}CD_3$-methionine (Cambridge Isotope Laboratories, Inc.). Cells were grown and passaged for over 7 days to ensure maximum synthesis of $^{13}CD_3$-S-adenosyl methionine (SAM) and subsequent maximum incorporation of this "heavy€ SAM into histone proteins as methylation sites [44]. Nuclei were isolated from harvested cell pellets using a nuclei isolation buffer (NIB) consisting of 0.4% nonidet P-40, 1 mM dithiothreitol, 10 μM microcystin, 10 mM sodium butyrate, and 300 μM 4-(2-aminoethyl) benzenesulfonyl fluoride hydrochloride [44]. *In nucleo* reactions were performed as described previously [45], with the following alterations. In these reactions, the reaction buffer consisted of the NIB buffer listed above (without the nonidet P-40 detergent) with the addition of 1 mM $^{12}CH_3$-SAM and 100 μM of either a control peptide (scrambled sequence) or inhibitor peptide (SQ037). The nuclei were incubated with this buffer for approximately 2 hours. No significant degradation of EZH2 methyltransferase was observed with or without the SQ037 inhibitor peptide treatment (Figure S6).

Histone Extraction and Mass Spectrometry Sample Preparation

Histones were extracted out of the nuclei with 10× volume 0.4N H_2SO_4 (incubated on ice for 2 hours), and precipitated from the acid supernatant with 25% final volume of trichloracetic acid. The precipitated histone pellet was washed once with 10 mL 0.1% HCl

Table 1. Sequence Selection, Fold Specificity, and Approximate Binding Affinity Results for Top 17 Peptides.

Run	Name	E	f_{spec}	K^*	13									Sequence											33
Run 1	*SQ010	216	10	1	G	K	A	P	R	K	Q	L	A	**S**	**A**	**K**	**K**	**A**	**A**	**A**	**T**	P	**R**	T	G
	*SQ011	428	11	2	G	K	A	P	R	K	Q	L	A	**A**	K	**S**	A	**A**	**A**	**K**	**T**	P	**R**	T	G
	SQ004	317	4	3	G	K	A	P	R	K	Q	L	A	**A**	**S**	**K**	A	**A**	**A**	**K**	**T**	P	**R**	T	G
	SQ003	248	3	4	G	K	A	P	R	K	Q	L	A	**S**	K	A	A	**A**	**A**	**K**	**T**	P	**R**	T	G
	SQ013	484	13	5	G	K	A	P	R	K	Q	L	A	**S**	**T**	**K**	**K**	**A**	**A**	**A**	**T**	P	**R**	**A**	G
	H3	-	-	6	G	K	A	P	R	K	Q	L	A	T	K	A	A	R	K	S	A	P	A	T	G
Run 2	*SQ020	438	7	1	G	K	A	P	R	K	Q	L	A	**S**	K	A	A	R	**A**	**A**	**T**	P	**K**	T	G
	*SQ025	494	12	2	G	K	A	P	R	K	Q	L	A	**A**	K	A	A	**T**	K	S	A	P	**R**	T	G
	SQ023	304	10	3	G	K	A	P	R	K	Q	L	A	T	K	A	**K**	**A**	**A**	S	A	P	**R**	T	G
	H3	-	-	4	G	K	A	P	R	K	Q	L	A	T	K	A	A	R	K	S	A	P	A	T	G
Run 3	*SQ035	372	10	1	G	K	A	P	R	K	Q	L	A	T	**S**	A	A	**W**	K	**P**	A	**R**	A	T	**K**
	*SQ032	299	7	2	G	K	A	P	R	K	Q	L	A	T	**P**	A	**W**	R	**A**	S	A	**K**	A	T	**K**
	*SQ026	363	1	3	G	K	A	P	R	K	Q	L	A	**P**	K	A	A	**T**	K	**N**	A	**R**	A	T	**S**
	SQ028	442	3	4	G	K	A	P	R	K	Q	L	A	**P**	**T**	A	A	**W**	K	S	A	**K**	A	T	**R**
	SQ031	266	6	5	G	K	A	P	R	K	Q	L	A	**P**	K	A	A	R	K	**N**	A	**T**	A	T	**S**
	H3	-	-	6	G	K	A	P	R	K	Q	L	A	T	K	A	A	R	K	S	A	P	A	T	G
Run 4	*SQ040	210	5	1	G	K	A	P	R	K	Q	L	A	**N**	**R**	**K**	**W**	**W**	K	**N**	**Y**	P	**R**	**D**	G
	*SQ043	306	8	2	G	K	A	P	R	K	Q	L	A	**R**	K	**N**	**W**	**W**	K	**N**	**Y**	P	**R**	**D**	G
	*SQ037	500	2	3	G	K	A	P	R	K	Q	L	A	**R**	**R**	**K**	**W**	**W**	**F**	**N**	**Y**	P	**Q**	**N**	G
	SQ041	16	6	4	G	K	A	P	R	K	Q	L	A	**R**	**N**	**K**	**W**	**W**	K	**N**	**Y**	P	**R**	**D**	G
	H3	-	-	5	G	K	A	P	R	K	Q	L	A	T	K	A	A	R	K	S	A	P	A	T	G

Sequence selection, fold specificity, and approximate binding affinity results for all 17 peptides ranked higher than native in approximate binding affinity calculation. Rankings are given for sequence selection (potential energy rank #1 = lowest potential energy, E), fold specificity (fold specificity rank #1 = highest specificity, f_{spec}), and approximate binding affinity (approximate binding affinity rank #1 = highest affinity, K^*). E and f_{spec} were not calculated for the native sequence. Mutations away from H3-peptide are indicated in bold. A * indicates a peptide that was submitted for experimental validation.

doi:10.1371/journal.pone.0090095.t001

in acetone, washed twice with 100% acetone, and allowed to air dry at room temperature. Histone proteins were then derivatized using propionic anhydride reagent as previously described [46], diluted with 50 µL of 100 mM ammonium bicarbonate, and digested with trypsin at a 10:1 protein:enzyme ratio with the reaction being quenched by the addition of 5 µL glacial acetic acid. Resultant histone peptides were then repropionylated a second time and the mixture was desalted using C18-based, homemade STAGE tips before mass spectrometry (MS) analysis as previously described [46].

NanoLC-MS/MS Based Quantitative Proteomics

Mass spectrometry (MS) analysis was performed as histone peptide digests were loaded onto fused silica capillary columns (ID: 75 µm) packed with Magic C18 reversed phase 5 µm resin particles (Michrom BioResources Inc.) by an Eksigent autosampler and nano-electrosprayed (2.5 kV) into an Orbitrap mass spectrometer (Thermo Scientific). Peptides were separated using reversed-phase high performance liquid chromatography (RP-HPLC) as previously described [44]. The Orbitrap instrument was operated in data-dependence mode as a full mass spectrum was acquired in the Orbitrap at 30000 resolution, followed by 7 data-dependent tandem mass spectra (MS/MS) acquired in the quadrupole ion trap. All MS/MS spectra were manually verified. Chromatographic peak integration was used to quantify the relative abundance of the various labeled and modified histone peptides as previously described [44,46].

Results

De Novo Design of Methyltransferase Inhibitors

The full *de novo* peptide design framework is described in detail in the Methods sections. It consists of three stages: an optimization-based sequence selection [19–23], fold specificity calculation [19,20,23,26], and approximate binding affinity calculation [24,25]. For the design of inhibitors of the lysine methyltransferase EZH2 target, four iterations of the method were run with different sets of biological constraints on the allowed sequences (see the Methods section for full details of each run). These runs were termed Run 1–4. The final set of candidate peptides consisted of 17 peptides predicted to have significantly higher binding affinity to EZH2 than the native H3-derived peptide. From these 17 peptides, 10 were chosen for experimental validation. The final set of peptides with higher calculated approximate binding affinity values is provided in Table 1. The selected peptides from each run are highlighted and ranked only in reference to the peptides in their respective sequence selection run. The full set of binding affinity results, exact values of the validation metrics, and the full ranking of the selected peptides over all runs are provided in Table S1.

Histone Methyltransferase and Mechanism of Action Enzymatic Assays

In order to assess the inhibitory capability of the candidate peptides experimentally, HMT enzymatic assays were conducted. These HMT assays assessed the EZH2-dependent transfer of

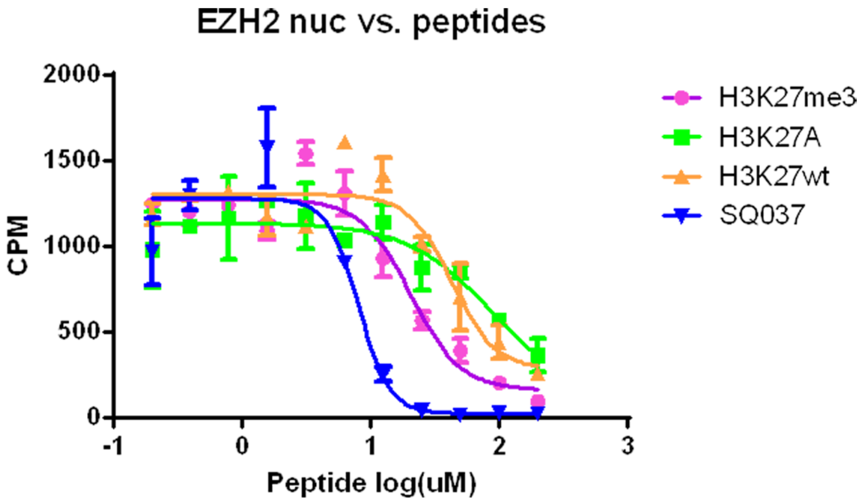

	H3K27me3	H3K27A	H3K27wt	SQ037
IC50	20.52	88.95	42.73	8.055

Figure 3. Designed Peptides Competitively Inhibit EZH2 Catalytic Activity. (A) A radiometric assay was used to determine the EZH2 catalytic activity in the absence (lane 1) or presence of 125 μM of candidate EZH2 inhibitor peptides (lanes 2–11). The inhibitory potential of native H3 peptide (lane 12) and an unrelated peptide (random; lane 13) was assessed. A reaction without peptide, but heat inactivated at 95°C for 5 min prior to incubation, was used to determine the background (lane 14). Shown is a fluorographic image of [3H]-labeled methyl groups incorporated on histone H3 (upper panel). Histones were visualized by Coomassie Blue staining (lower panel). (B) A high throughput radiometric assay was used to determine the inhibitory potential of candidate peptides. Shown is the absolute EZH2 HMT activity (counts per minute, cpm). (C,D) The catalytic activity of EZH2(C) and EZH1(D) was assessed in the absence (lane 2) or presence (lane 3) of SQ037 [125 μM]. Shown is a fluorographic image of [3H]-labeled methyl groups incorporated on histone H3 (upper panel). Histones and PRC2 constituents were visualized by Coomassie Blue staining (lower panel).
doi:10.1371/journal.pone.0090095.g003

tritiated methyl-groups from the methyl-donor SAM to reconstituted oligonucleosomes (see Methods for details). First, candidate peptides were inspected in endpoint assays with a final peptide concentration of 125 μM. Most of the peptides were identified as weak inhibitors of EZH2 (Figure 3.A). However, peptide SQ037 showed significant suppression of EZH2 catalytic activity (Figure 3.A, lane 11) that was superior to the inhibitory potential of the native H3K27 peptide (Figure 3.A, lane 12). To corroborate and expand on these experimental findings, a more sensitive high throughput assay was implemented that relied on streptavidin-based capture of biotinylated oligonucleosomes and scintillation counting in a 384-well format (see Methods for details). Using this assay, SQ037 was confirmed as the most potent among the tested inhibitors. Importantly, since this assay was carried out under balanced conditions (specifically, at K_m for substrate) several other peptides showed significant inhibition of EZH2 (Figure 3.B). Moreover, SQ037 inhibited both PRC2 complexes reconstituted with either EZH2 or its homolog EZH1 (Figures 3.C–D).

To quantitatively measure the inhibition properties of the designed sequences, peptide dose titrations were performed. The concentration of peptide required to suppress 50% of the enzymatic activity (i.e. half-maximal inhibitory concentration, IC_{50}) and the Hill coefficient (measure of cooperativity of binding) were calculated (Table 2). The previously identified peptide, SQ037, remained the most potent peptide, with an approximate IC_{50} of 13.57 μM. While significantly higher than previously discovered small molecule inhibitors, this level of potency is the first observed for computationally design peptides targeting EZH2 and shows the potential use and development of the peptidic inhibitor as a chemical probe in future EZH2 biological investigations. For reference, the IC_{50} for the small molecule inhibitor EI1 is approximately 15 nM [16].

The aim of the study was to develop inhibitors for the interrogation of chromatin biology, as well as show that the peptide design framework presented can produce specific peptides for methyltransferase inhibition. In pursuit of both these goals it is important not only to demonstrate inhibitory potential, but to understand the mechanism of action of the peptidic inhibitor. Understanding the mechanism of action allows us to determine whether the competitive inhibition targeted by the design framework and the input biological constraints has been successful. In order to show that the candidate peptide, SQ037, inhibits the substrate binding competitively, HMT assays were carried out in the presence of increased enzyme, cofactor, and substrate concentrations (Figure 4). While 10-fold enzyme and SAM did not significantly alter the inhibitory potential of SQ037 (Figure 4.A and 4.B, respectively), a 10-fold increase in substrate shifted the IC_{50} approximately 5-fold (Figure 4.C), suggesting that the binding of SQ037 is competitive with the substrate.

Finally, several further studies were performed in order to assess whether the top designed peptide performed better than a simple point mutation of the lysine targeted for methylation. Since there is little experimental evidence for which mutation should be chosen for the comparative HMT enzymatic assays, a simple alanine mutation, K27A, was chosen to test against. The results of the HMT enzymatic assays are provided in Figure 5. These results both confirm that the top candidate peptide, SQ037, is significantly more potent than the native peptide and demonstrate higher potency than the K27A mutation. This is a strong confirmation of the success of the design method, which is capable

of designing a peptide outside the potency of what could be expected by rational design alone.

In nucleo Methyltransferase Inhibition Reactions Using Quantitative Mass Spectrometry

Encouraged by the positive *in vitro* results, experiments were designed to test if the top computationally designed inhibitor peptide elicited the same effect in a cell-based setting. As larger molecules such as peptides are typically more difficult to permeate through outer cell membranes, purified nuclei were used to determine if naturally produced EZH2 is inhibited by SQ037 as well. Such a system takes into account binding partners to the PRC2 complex, most likely resulting in more active enzymes, and a chromatin substrate that is more representative of the actual *in vivo* higher order structures. SAM content within the nuclei, however, is diluted, requiring SAM supplement to the reaction buffer. The experimental design is depicted in Figure 6. Cells were grown in $^{13}CD_3$-methionine for over a week to allow for near 100% fully labeled generation of $^{13}CD_3$-S-adenosyl methionine (SAM), which were incorporated into histones as methyl groups [44]. Greater than 98% labeling efficiency of most histone methylation sites was generally detected using this approach (data not shown). Using these nuclei as the reaction template (containing the histone-modifying enzyme complexes and histone substrate proteins), unlabeled "light€ SAM was added along with either a scrambled sequence control or an inhibitor peptide and the nuclei were incubated in the buffer for 2 hours. Previously methylated histone sites would all be "heavy" labeled, while newly methylated

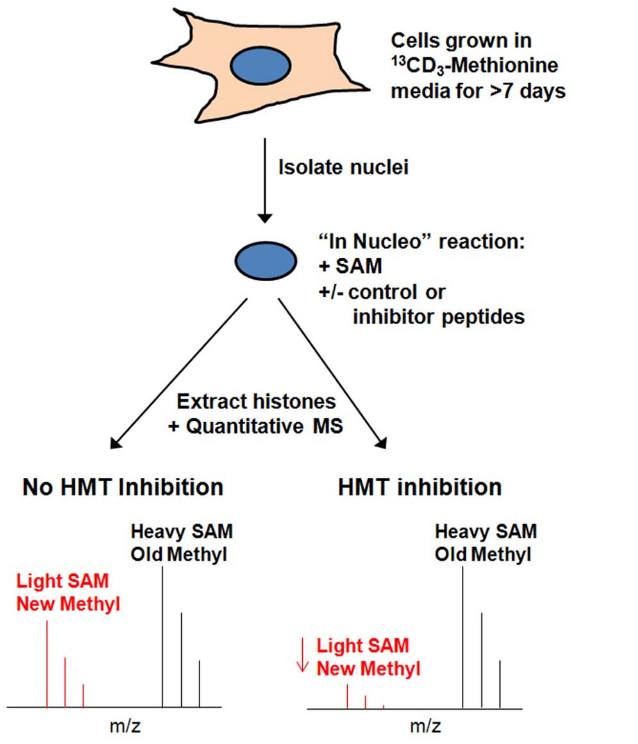

Figure 4. Mechanism of Action Enzymatic Assay Results. (A) Peptide dose response curve in the EZH2 HMT assay using 100 nM (10× enzyme) and 10 nM (1×enzyme) of reconstituted, recombinant PRC2. (B) Peptide dose response curve in the EZH2 HMT assay using 2.4 μM (10× SAM) and 0.24 μM (1× SAM) of 3H-labeled SAM. (C) Peptide dose response curve in the EZH2 HMT assay using 60 μM (10× substrate) and 6 μM (1× substrate) of H3K27me1.
doi:10.1371/journal.pone.0090095.g004

sites would all be "light€ labeled. This *in nucleo* assay monitored the effect that the control or inhibitor peptides exhibited on newly methylated histone sites and hence how they affected HMT activity. If the peptide had an inhibitory effect on the function of a particular histone methyltransferase, then the addition of new ("light") methyl groups to the histone sites would be reduced in comparison to a control peptide with no inhibitory effect. As a result, the ratio of old ("heavy") to new ("light") methylated histone sites produced with the addition of an inhibitory peptide would be reduced in comparison to the ratio produced with the addition of a control peptide with no inhibitory effect.

A snapshot of these experiments is shown in Figure 7.A-F. Mass spectra are shown of the 27–40 residue peptide from canonical histone H3 containing the K27 trimethylation site (KSA-PATGGVKKPHR) from the *in nucleo* reactions with (A) SAM only, (B) SAM plus the scrambled sequence control peptide and (C) SAM plus the SQ037 inhibitor peptide. The peak at 552.680 m/z corresponds to the $[M+3H]^{3+}$ ion that is the "heavy"-labeled old H3K27me3 peptide species. The old H3K27me3 is separated by 4 m/z (12.062 Da/3+charge = 4 m/z shift) from the "light"-labeled new H3K27me3 species (548.658 m/z) that represents the newly synthesized methylation mark. As this *in nucleo* assay is only performed for a relatively short time, only a small amount (<3%) of new H3K27 trimethylation was generated when only "light" SAM is added (Figure 7.A), consistent with the fairly slow dynamics of most histone methylation sites [44]. This amount of new H3K27 trimethylation was not inhibited when using the scrambled sequence control (Figure 7.B), but was noticeably lower when the SQ037 inhibitor peptide was used (Figure 7.C). The amount of old and new histone methylation was quantified from the *in nucleo* assay [44] for the reactions where only SAM was added, SAM and control peptide were added, and SAM and the inhibitor SQ037 peptide were added (Figures 7.D–F). Statistically significant differences in the abundance levels were found for the newly generated H3K27me2 (*$P<0.002$) and H3K27me3 (*$P<0.0001$) peptides for the reactions where the SQ037 inhibitor peptide was added compared to the reactions where only SAM (Figure 7.D) or the scrambled control sequence was added (Figure 7.E). Although this difference was more pronounced for the H3K27me3 form than the H3K27me2 peptide, both forms are known to be products of the targeted histone methyltransferase EZH2. H3K27me1 was not found to substantially decrease in abundance under any conditions. This is consistent with previous studies indicating that EZH2 may not contribute to the creation of this degree of methylation [47].

The specificity of SQ037 was tested through the examination of the abundance levels of other histone methylation sites that are not substrates for EZH2 under the above mentioned conditions. The levels of new methylation at these non-EZH2 targeted sites were not altered, such as is shown for H3K9me3 (Figure 7.F), a modification site targeted by SUV39H1 and other enzymes [1]. A full list of histone methylation sites that were not altered by the addition of SQ037 is provided in Table S2. These data suggest that the SQ037 inhibitor peptide is specific in its ability to interfere with EZH2 mediated H3K27 methylation in a more physiological setting. The specificity of an inhibitor is one of the major properties of an effective chemical probe [18,48]. The fact that the methylation of H3K27 is significantly inhibited in comparison to many other histone methylation sites provides confidence in the capability of the peptide design framework to create peptidic inhibitors to study chromatin biology.

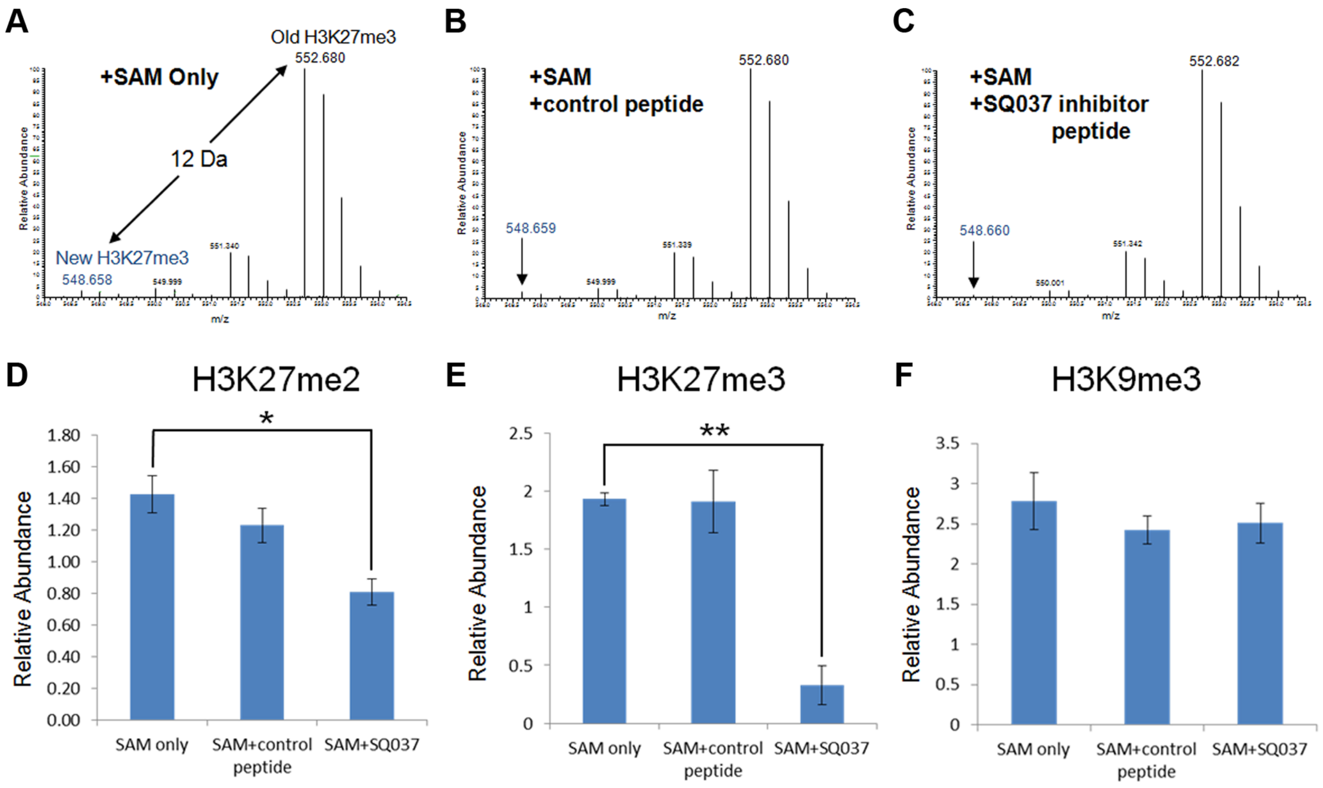

Figure 5. Potency of Top Designed Peptide vs. Native and Mutated Peptides. (A) Peptide dose response curve in the EZH2 HMT assay using 100 nM (10× enzyme) and 10 nM (1×enzyme) of reconstituted, recombinant PRC2. (B) Peptide dose response curve in the EZH2 HMT assay using 2.4 μM (10× SAM) and 0.24 μM (1× SAM) of 3H-labeled SAM. (C) Peptide dose response curve in the EZH2 HMT assay using 60 μM (10× substrate) and 6 μM (1× substrate) of H3K27me1.
doi:10.1371/journal.pone.0090095.g005

Discussion

The aim of this study was to use a computational *de novo* peptide design method to design peptidic inhibitors of the methyltransferase enzyme EZH2. Due to the specificity that peptidic inhibitors have towards their binding partners, the designed peptides in this study have potential application as chemical probes in the interrogation of chromatin biology. In order to produce quality chemical probes, one must satisfy several important characteristics [48]. These include proven potency, specificity, and known mechanism. For this reason, the experimental validation of the peptides in this study focused on these three areas. *In vitro* tests demonstrated potent inhibitory properties of several of the computationally designed candidate peptides, the best being SQ037 with an IC_{50} of 13.57 μM. This is the first study that utilizes a computational design method to discover peptides that directly inhibit EZH2. Such potency and selectivity must also be demonstrated *in vivo*. Due to the size of the peptides designed, cell permeability could be unlikely. For this reason an *in nucleo* methyltransferase inhibition experiment using quantitative mass spectrometry was developed to demonstrate that the designed peptides elicited the same effects in a cell-based setting. This is a step towards *in vivo* testing of the peptides, however further truncation and modification of the peptides would be needed to produce cell permeable peptidic inhibitors. These experiments showed that in the presence of the most potent peptide from the *in vitro* testing, SQ037, the production of methylated H3K27 was significantly reduced. Furthermore, testing was done using several other histone lysine modification sites, such as H3K9, to show that the most potent peptide demonstrated specificity *in nucleo*. These tests show that the predicted peptides retain their inhibitory properties *in nucleo*, as well as demonstrate specificity to EZH2 inhibition. Finally, a mechanism of action assay was performed in order to show the mechanism of action for the inhibitors. The most potent peptide was shown to be substrate-competitive. The experimental validation tests demonstrated favorable properties of the designed peptides for their potential use as chemical probes.

Table 2. Experimental IC_{50} and Hill coefficients for designed inhibitors of EZH2.

Name	IC_{50} (μM)	Hill Coeff.
SQ010	54.89±1.26	0.819
SQ011	>200	-
SQ020	34.21±1.46	0.549
SQ025	>200	-
SQ035	>200	-
SQ032	>200	-
SQ026	19.58±1.12	1.374
SQ040	41.02±1.19	0.912
SQ043	39.70±1.17	1.143
SQ037	13.57±1.24	0.704

Experimental IC_{50} and Hill coefficients for designed inhibitors of EZH2.
doi:10.1371/journal.pone.0090095.t002

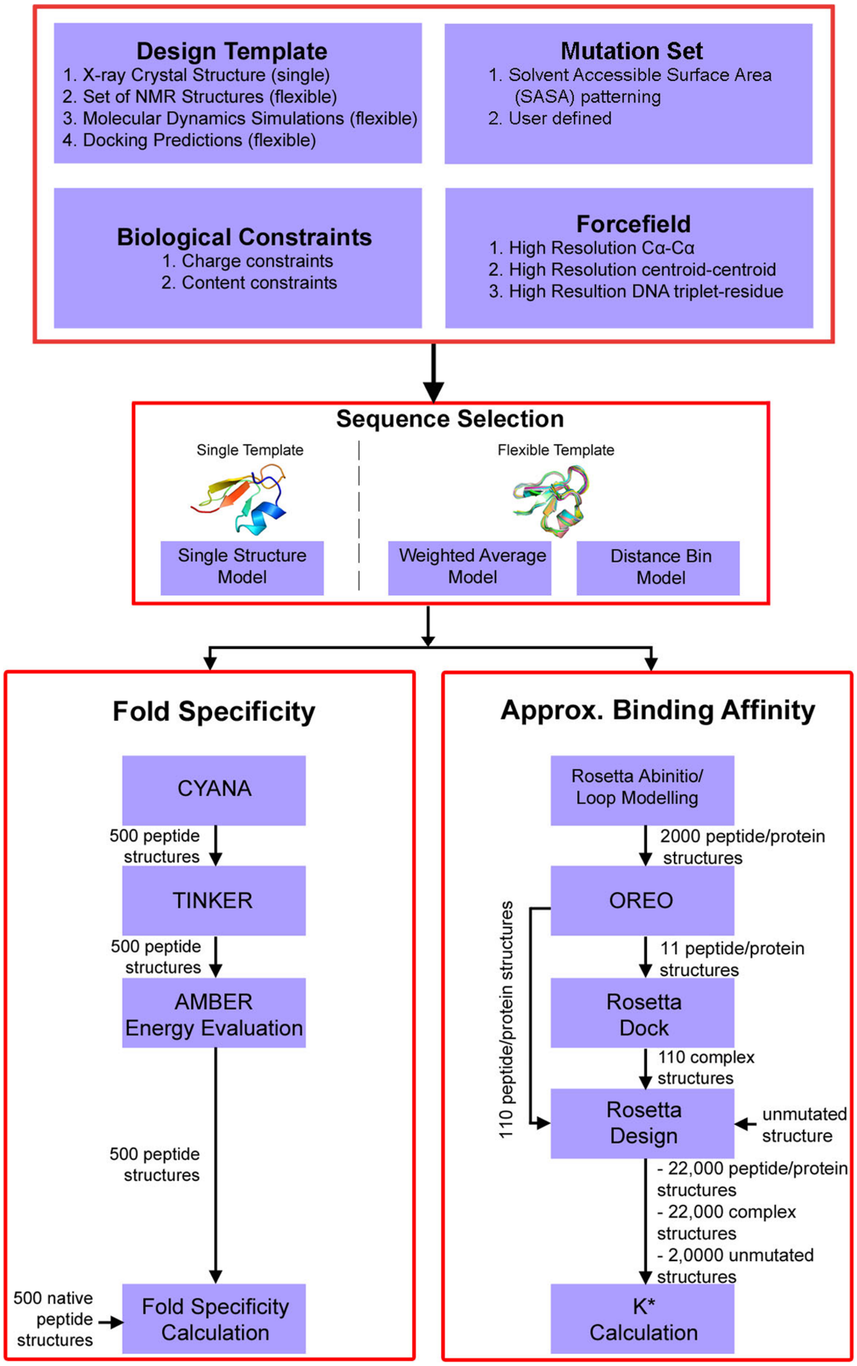

Figure 6. Schematic of *In nucleo* Experiments. Schematic describing the in nucleo HMT inhibition experiments with expected results for HMT inhibition and no HMT inhibition.
doi:10.1371/journal.pone.0090095.g006

In order to demonstrate the potential of the design method in a broader context, the top designed peptide, SQ037, was compared to a simple, rationally designed point mutation, K27A. The results show that the K27A mutant had an IC_{50} nearly an order of magnitude larger than the designed peptide in the *in vitro* experiments. This confirms the success of the *de novo* design method, as it is capable of outperforming a simple rationally designed peptide inhibitor. It should be noted that the IC_{50} for the K27A peptide calculated in this study differs from that reported in literature [15]. This is most likely due to variability in the experimental conditions that make the comparison of IC_{50} values across studies difficult. This is the reason why the K27A mutant was synthesized independently in order to make an accurate comparison to the designed peptides.

The full set of experimental results demonstrate the applicability of the computational design method to the development of histone-modifying enzyme inhibitors. This is an important advancement, as the computational method is capable of expanding the sequence space search in comparison to traditional experimental peptide design methods through the use of optimi-

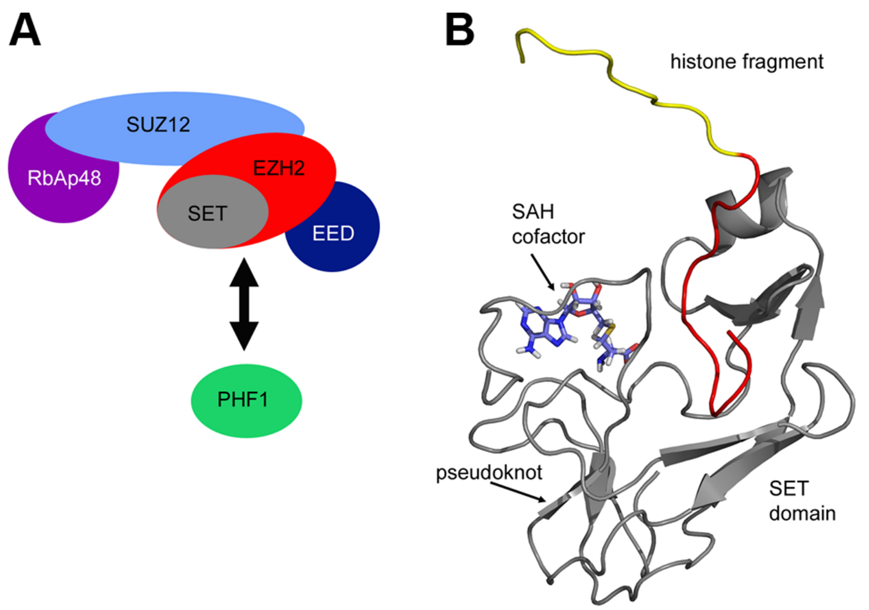

Figure 7. ***In nucleo*** **Experiments to Test Effects of the Inhibitor Peptide on HMT Activity.** Quantitative proteomics distinguish newly generated "heavy" histone methylation at H3K27 from old, "light" methylation. MS monitoring of new H3K27me3 from the in nucleo reaction with (A) SAM only, (B) SAM plus the scrambled control peptide added, and (C) SAM plus SQ037 inhibitor peptide added. Relative abundance levels across all conditions for (D) H3K27me2, (E) H3K27me3 and (F) H3K9me3 are shown. Statistical significance between abundance levels is indicated, *$P<0.002$ and **$P<0.0001$. Three biological replicates were used. Note that the specified 12 Da shift is nominal, exact value is 12.062 Da.
doi:10.1371/journal.pone.0090095.g007

zation techniques. While this study presents a specific inhibitor of a single lysine methyltransferase of biological relevance, EZH2, the changes to the method necessary to design inhibitors of other histone-modifying enzymes are minimal and worthy of discussion. The changes necessary primarily concern the structural template chosen for design. A relevant structural template of the desired protein target is needed for any application of the protein design method. This is a key aspect of the method, as this template is used in all three stages of the design. It is generally desirable to have either an NMR or crystallographic structure of the target protein. However, this study demonstrates the successful design of inhibitors of EZH2 through the use of a low-homology vSET structure that also binds H3K27. This suggests that the design of such histone-modifying enzymes may not need an exact experimental structure, but rather a structure of a protein that binds the same substrate. Perhaps the specific binding interactions necessary for design are conserved across enzymes that modify identical sites. If so, this would allow structure based design methods to target a wider range of enzymatic targets than previously thought.

It is also important to retrospectively analyze the biological constraints used in the study to see if there are trends that may be important for future designs. There were four sets of biological constraints used in this study: fixed P30 and G33 with sequence rearrangement only, fixed P30 and G33 with up to five rearrangements, up to five mutations, and fixed P30 and G33 with an upper bound of two on the number of each amino acid type. All sets maintained the native charge of +3. From the endpoint assay results presented in Figure 3.A, SQ037, SQ040, and SQ043 stood out qualitatively in comparison to control. The quantitative inhibitory assays and IC_{50} results (Figure 3.B and Table 2) confirmed that these three peptides were among the best designed inhibitors. All three of the peptides were derived from Run 4 of the Sequence Selection stage. Comparing the sequences from Run 4 to the other runs, a distinct charge feature of the sequences can be identified. While all the runs were forced to maintain the native charge of +3 on the designed peptides, Run 4 had loose mutational constraints that allowed for the addition of pairs of negatively and positively charged amino acids. This resulted in a higher charge content in the sequences, especially in positions 22–25, without a change in overall charge. This charge feature may be important for EZH2 inhibition and could be used to guide future inhibitor design. The designed peptide SQ026 from Run 3 also deserves some analysis as it is the only successful design where P30 and G33 mutations were tolerated and had the second lowest IC_{50} value after SQ037. Analyzing this sequence in reference to the other unsuccessful peptide designs from Run 3 and the successful designs from Run 4, a consistent mutation of S28N is observed for successful inhibitor design. This could be an important mutation for inhibition and perhaps is the reason that SQ026 tolerated mutations in P30 and G33, while SQ035 and SQ032 did not. This would also explain why Run 1 and Run 2 were less successful in their design. Not only does the more restrictive constraints not allow for the increase in charge constraints seen in Run 4, but since there are no asparagine residues in the initial sequences, there was no opportunity for the S28N mutation through simple rearrangement. This suggests that the constraints used for Runs 1 and 2 were perhaps too restrictive and should be loosened in a similar manner to Run 3 and Run 4 for future designs. Overall, from this analysis it would seem that Run 4 contains the optimum set of constraints for this design, allowing for both increased charge content and the S28N mutations, while restricting changes to positions 30 and 33 that were largely unsuccessful. While the S28N mutation may be specific to EZH2 inhibitor design, the increased charge constraint may be characteristic of general histone-modifying enzyme inhibitor design and is worthy of further exploration.

Analysis of the template-based constraints demonstrate how one can use the results from this study to guide future EZH2 and other histone-modifying enzyme design. In order to guide future peptide inhibitor design more generally, however, one must analyze the

influence of the fold specificity and approximate binding affinity metrics on the capability of the method to correctly identify peptidic inhibitors. Hence, it is useful to focus on the peptides derived from Run 4 (SQ037, SQ040, and SQ043) that stood out in the endpoint assay and IC_{50} results. In analyzing the quantitative results from the Fold Specificity and Approximate Binding Affinity validation stages (Table S1), these three peptides are the top three ranked peptides in Fold Specificity and three of the top four in Approximate Binding Affinity. Since these values are used simply as a ranking metric, this demonstrates the usefulness that both metrics have in producing designed peptides with a high probability of success.

Besides analyzing the influence of input biological constraints and the selection metrics, it is also important to analyze the results of the method from a structural perspective. This allows us to determine how well the validation stages predict consistent contacts that may play an important role in the inhibitory properties of the peptides. Such knowledge is important for future design studies. Taking the four lowest energy structures from the docking runs for the top peptide SQ037, the contacts observed consistently across the four structures were identified (Figures S1, S2, S3, S4). The PDB files used in the analysis are provided in File S1 (structures.zip). The analysis of the structures reveal three distinct contacts (shown in Figure S5), found in all or most of the four structures. These include a contact between K24 of the peptide and D41 of the protein, W26 of the peptide and M57 of the protein, and N32 of the peptide and D108/Y109. While all of these contacts are interesting in interpreting the results of the study, the contact between K24 and D41 is intriguing in two regards. The first is that this constitutes a potential salt bridge, previously unexploited in the native structure. Further, the D41 amino acid is a position of conservation between the human and viral SET domains. Such novel contacts to the conserved binding site residues may help determine which amino acid positions in the designed sequence are important for antagonistic binding.

Further analysis of the mutation constraints can be performed using structural analysis. The allowed mutations in each position are chosen based on the Solvent Accessible Surface Area (SASA) of the template structure. This is such that buried polar groups would have the opportunity to mutate to hydrophobic residues that may fit the structural environment better. In the successful design from Run 4, there was one interesting position that mutated from a buried charged amino acid to a hydrophobic amino acid consistently, R26W. As mentioned previously, W26 is an interesting position due to its novel interactions with the hydrophobic M57 position in the structure of the top inhibitor, SQ037. However, upon visual inspection of the SQ037 structure, the interaction occurs primarily between the side-chain atoms of W26 and the main-chain atoms of M57. More interestingly, adjacent to the M57 position there is a negatively charged lysine residue (shown in Figure S5.B) that could have unfavorable interactions with the native arginine. The mutation to the tryptophan may be important in preventing these unfavorable interactions as the peptidic inhibitor approaches and binds to the enzyme, thus improving the overall favorability of binding. This analysis provides an example where the constraints allowing for the mutation from a buried polar group to a hydrophobic group not only resulted in potentially favorable hydrophobic-hydrophobic interactions, but also prevented undesirable native charge-charge interactions. This finding shows the benefit of these SASA-based mutation constraints, which can be generally produced for any application of the peptide inhibitor design method.

Supporting Information

Figure S1 Contact Map for a Top Bound Structure of SQ037, cd2G46_ppk.0383.pdb. Contact map for one of the top bound structures produced for the top designed inhibitor SQ037, cd2G46 ppk.0383.pdb. All protein position numbers correspond to the numbering given in PDB:2G46. All peptide position numbers correspond to the numbering used in Table 1. Distances are given in Å, and only contacts between 4 Å–10 Å are visualized.
(TIF)

Figure S2 Contact Map for a Top Bound Structure of SQ037, cd2G46_ppk.0514.pdb. Contact map for one of the top bound structures produced for the top designed inhibitor SQ037, cd2G46 ppk.0514.pdb. All protein position numbers correspond to the numbering given in PDB:2G46. All peptide position numbers correspond to the numbering used in Table 1. Distances are given in Å, and only contacts between 4 Å–10 Å are visualized.
(TIF)

Figure S3 Contact Map for a Top Bound Structure of SQ037, cd2G46_ppk.1010.pdb. Contact map for one of the top bound structures produced for the top designed inhibitor SQ037, cd2G46 ppk.1010.pdb. All protein position numbers correspond to the numbering given in PDB:2G46. All peptide position numbers correspond to the numbering used in Table 1. Distances are given in Å, and only contacts between 4 Å–10 Å are visualized.
(TIF)

Figure S4 Contact Map for a Top Bound Structure of SQ037, cd2G46_ppk.1330.pdb. Contact map for one of the top bound structures produced for the top designed inhibitor SQ037, cd2G46 ppk.1330.pdb. All protein position numbers correspond to the numbering given in PDB:2G46. All peptide position numbers correspond to the numbering used in Table 1. Distances are given in Å, and only contacts between 4 Å–10 Å are visualized.
(TIF)

Figure S5 Contact Highlights for the Bound Structure of Sequence SQ037. Low energy structure for SQ037 with several important protein (blue) and peptide (green) positions labelled. Different angles are provided to highlight contacts with peptide positions (A) K24, (B) W26, and (C) N32.
(TIF)

Figure S6 Western Blot EZH2 Degradation Experiments. Western Blot analysis comparing levels of human EZH2 (¼ 98 kD, Cell Signaling) and human lamin B1 (¼ 66 kD, Invitrogen) from extracts of in nucleo reactions containing or lacking the SQ037 inhibitor peptide.
(TIF)

Table S1 Results for Sequences Tested by Approximate Binding Affinity Validation. Rankings and exact calculated values are given for sequence selection (potential energy rank #1 = lowest potential energy, E), fold specificity (fold specificity rank #1 = highest specificity, f_{spec}), and approximate binding affinity (approximate binding affinity rank #1 = highest affinity, K^*). E and f_{spec} were not calculated for the native sequence. * indicated peptide tested experimentally.
(DOCX)

Table S2 Relative Abundance of All Peptides Corresponding to a Given Methylated State. Relative abundance of all peptides

corresponding to a given methylated state containing at least one unlabeled $^{12}CH_3$-methyl group from in nucleo reactions performed with 100 μM control or SQ037 peptide. Thus, for H3K27me3, the relative abundance corresponds to (H3K27me3:0+H3K27me3:1+H3K27me3:2)/(H3K27me3:0+H3K27me3:1+H3K27me3:2+H3K27me3:3). H3K9me1 corresponds to the monomethylated 9–17 H3 peptide (KSTGGKAPR), H4K20me1 and me2 correspond to the 20–23 H4 peptide (KVLR) monomethylated and dimethylated on K20 respectively, H3K36me1 and H3K36me2 correspond to the 27–40 H3 peptide (KSAPATGGVKKPHR) monomethylated and dimethylated on K36 respectively, and H3K79me1 and H3K79me2 correspond to the 73–83 H3 peptide (EIAQDFKTDLR) monomethylated and dimethylated on K79 respectively.
(DOCX)

File S1 Structures.zip. Structure files for top bound structures produced for the top designed inhibitor SQ037. Four structures are included: cd2G46_ppk.1330.pdb, cd2G46_ppk.1010.pdb, cd2G46_ppk.0514.pdb, and cd2G46_ppk.0383.pdb. These structures were used in contact analysis for the top designed inhibitor, SQ037. All structures are provided in .pdb format with protein position numbering corresponding to the numbering given in PDB:2G46.
(ZIP)

Author Contributions

Conceived and designed the experiments: JS MBP CAF SG CL PT BMZ AM BAG. Performed the experiments: JS MBP SG CL BMZ AM. Analyzed the data: JS MBP CAF AG SG CL PT BMZ AM BAG. Contributed reagents/materials/analysis tools: JS MBP CAF AG SG CL PT BMZ AM BAG. Wrote the paper: JS MBP SG BMZ AM CL AG PT BAG CAF.

References

1. Shi Y, Whetstine JR (2007) Dynamic regulation of histone lysine methylation by demethylases. Mol Cell 25: 1–14.
2. Stavropoulos P, Blobel G, Hoelz A (2006) Crystal structure and mechanism of human lysine-specific demethylase-1. Nat Struct Mol Biol 13: 626–632.
3. Khoury GA, Baliban RC, Floudas CA (2011) Proteome-wide post-translational modification statistics: frequency analysis and curation of the swiss-prot database. Sci Rep 1.
4. Mund C, Lyko F (2010) Epigenetic cancer therapy: Proof of concept and remaining challenges. BioEssays 32: 949–957.
5. Varambally S, Dhanasekaran SM, Zhou M, Barrette TR, Kumar-Sinha C, et al. (2002) The polycomb group protein EZH2 is involved in progression of prostate cancer. Nature 419: 624–629.
6. Kleer CG, Cao Q, Varambally S, Shen R, Ota I, et al. (2003) EZH2 is a marker of aggressive breast cancer and promotes neoplastic transformation of breast epithelial cells. P Natl Acad Sci USA 100: 11606–11611.
7. Sellers WR, Loda M (2002) The EZH2 polycomb transcriptional repressora marker or mover of metastatic prostate cancer? Cancer Cell 2: 349–350.
8. Simon JA, Lange CA (2008) Roles of the EZH2 histone methyltransferase in cancer epigenetics. Mutat Res-Fund Mol M 647: 21–29.
9. McCabe MT, Ott HM, Ganji G, Korenchuk S, Thompson C, et al. (2012) EZH2 inhibition as a therapeutic strategy for lymphoma with EZH2-activating mutations. Nature 492: 108–112.
10. Gonzalez ME, Li X, Toy K, DuPrie M, Ventura AC, et al. (2008) Downregulation of EZH2 decreases growth of estrogen receptor-negative invasive breast carcinoma and requires BRCA1. Oncogene 28: 843–853.
11. Spannhoff A, Sippl W, Jung M (2009) Cancer treatment of the future: Inhibitors of histone methyltransferases. Int J Biochem Cell B 41: 4–11.
12. Tan J, Yang X, Zhuang L, Jiang X, Chen W, et al. (2007) Pharmacologic disruption of polycombrepressive complex 2-mediated gene repression selectively induces apoptosis in cancer cells. Gene Dev 21: 1050–1063.
13. Fiskus W, Buckley K, Rao R, Mandawat A, Yang Y, et al. (2009) Panobinostat treatment depletes EZH2 and DNMT1 levels and enhances decitabine mediated de-repression of JunB and loss of survival of human acute leukemia cells. Cancer Biol Ther 8: 939–950.
14. Choudhury SR, Balasubramanian S, Chew YC, Han B, Marquez VE, et al. (2011) (−)- Epigallocatechin-3-gallate and DZNep reduce polycomb protein level via a proteasome-dependent mechanism in skin cancer cells. Carcinogenesis 32: 1525–1532.
15. Diaz E, Machutta CA, Chen S, Jiang Y, Nixon C, et al. (2012) Development and validation of reagents and assays for EZH2 peptide and nucleosome high-throughput screens. J Biomol Screen 17: 1279–1292.
16. Qi W, Chan H, Teng L, Li L, Chuai S, et al. (2012) Selective inhibition of EZH2 by a small molecule inhibitor blocks tumor cells proliferation. P Natl Acad Sci USA 109: 21360–21365.
17. Knutson SK, Wigle TJ, Warholic NM, Sneeringer CJ, Allain CJ, et al. (2012) A selective inhibitor of EZH2 blocks H3K27 methylation and kills mutant lymphoma cells. Nat Chem Biol 8: 890–896.
18. Luo M (2012) Current chemical biology approaches to interrogate protein methyltransferases. ACS Chem Biol 7: 443–463.
19. Klepeis JL, Floudas CA, Morikis D, Tsokos CG, Argyropoulos E, et al. (2003) Integrated structural, computational and experimental approach for lead optimization: Design of compstatin variants with improved activity. J Am Chem Soc 125: 8422–8423.
20. Klepeis JL, Floudas CA, Morikis D, Tsokos CG, Lambris JD (2004) Design of peptide analogs with improved activity using a novel de novo protein design approach. Ind Eng Chem Res 43: 3817–3826.
21. Fung HK, Rao S, Floudas CA, Prokopyev O, Pardalos PM, et al. (2005) Computational comparison studies of quadratic assignment like formulations for the in silico sequence selection problem in de novo protein design. J Comb Optim 10: 41–60.
22. Fung HK, Taylor MS, Floudas CA (2007) Novel formulations for the sequence selection problem in de novo protein design with flexible templates. Optim Method Softw 22: 51–71.
23. Fung HK, Floudas CA, Taylor MS, Zhang L, Morikis D (2008) Toward full-sequence de novo protein design with flexible templates for human beta-defensin-2. Biophys J 94: 584–599.
24. Bellows ML, Fung HK, Floudas CA, López de Victoria A, Morikis D (2010) New compstatin variants through two de novo protein design frameworks. Biophys J 98: 2337–2346.
25. Bellows ML, Taylor MS, Cole PA, Shen L, Siliciano RF, et al. (2010) Discovery of entry inhibitors for HIV-1 via a new de novo protein design framework. Biophys J 99: 3445–3453.
26. Bellows-Peterson ML, Fung HK, Floudas CA, Kieslich CA, Zhang L, et al. (2012) De novo peptide design with C3a receptor agonist and antagonist activities: Theoretical predictions and experimental validation. J Med Chem 55: 4159–4168.
27. Qian C, Wang X, Manzur K, Sachchidanand, Farooq A, et al. (2006) Structural insights of the specificity and catalysis of a viral histone H3 lysine 27 methyltransferase. J Mol Biol 359: 86–96.
28. Hubbard SJ, Thornton JM (1993) Naccess version 2.1.1. Computer Program, Department of Biochemistry and Molecular Biology, University College London.
29. Rajgaria R, McAllister SR, Floudas CA (2008) Distance dependent centroid to centroid force fields using high resolution decoys. Proteins 70: 950–970.
30. Guntert P, Mumenthaler C, Wuthrich K (1997) Torsion angle dynamics for NMR structure calculation with the new program DYANA. J Mol Biol 273: 283–298.
31. Guntert P (2004) Automated NMR structure calculation with CYANA. Methods Mol Biol 278: 353–378.
32. Ponder J (1998) TINKER, software tools for molecular design. 1998. Department of Biochemistry and Molecular Biophysics, Washington University School of Medicine: St. Louis, MO.
33. Cornell WD, Cieplak P, Bayly CI, Gould IR, Merz KM, et al. (1995) A 2nd generation force-field for the simulation of proteins, nucleic-acids, and organic-molecules. J Am Chem Soc 117: 5179–5197.
34. Lilien RH, Stevens BW, Anderson AC, Donald BR (2005) A novel ensemble-based scoring and search algorithm for protein redesign and its application to modify the substrate specificity of the gramicidin synthetase a phenylalanine adenylation enzyme. J Comput Biol 12: 740–761.
35. Lee MR, Baker D, Kollman PA (2001) 2.1 and 1.8 Å Cα RMSD structure predictions on two small proteins, HP-36 and S15. J Am Chem Soc 123: 1040–1046.
36. Rohl CA, Baker D (2002) De novo determination of protein backbone structure from residual dipolar couplings using rosetta. J Am Chem Soc 124: 2723–2729.
37. Rohl CA, Strauss CEM, Misura KMS, Baker D (2004) Protein structure prediction using rosetta. Method Enzymol 383: 66–93.
38. DiMaggio PA, McAllister SR, Floudas CA, Feng XJ, Rabinowitz JD, et al. (2008) Biclustering via optimal re-ordering of data matrices in systems biology: Rigorous methods and comparative studies. BMC Bioinformatics 9: 458.
39. DiMaggio PA, McAllister SR, Floudas CA, Feng XJ, Rabinowitz JD, et al. (2010) A network flow model for biclustering via optimal re-ordering of data matrices. J Global Optim 47: 343–354.
40. Daily MD, Masica D, Sivasubramanian A, Somarouthu S, Gray JJ (2005) CAPRI rounds 3–5 reveal promising successes and future challenges for RosettaDock. Proteins 60: 181–186.

41. Gray JJ, Moughon S, Wang C, Schueler-Furman O, Kuhlman B, et al. (2003) Protein-protein docking with simultaneous optimization of rigid-body displacement and side-chain conformations. J Mol Biol 331: 281–299.
42. Gray JJ, Moughon SE, Kortemme T, Schueler-Furman O, Misura KMS, et al. (2003) Proteinprotein docking predictions for the CAPRI experiment. Proteins 52: 118–122.
43. Kuhlman B, Baker D (2000) Native protein sequences are close to optimal for their structures. P Natl Acad Sci USA 97: 10383–10388.
44. Zee BM, Levin RS, Xu B, LeRoy G, Wingreen NS, et al. (2010) In vivo residue-specific histone methylation dynamics. J Biol Chem 285: 3341–3350.
45. Fischle W (2005) In nucleo enzymatic assays for the identification and characterization of histone modifying activities. Methods 36: 362–367.
46. Plazas-Mayorca MD, Zee BM, Young NL, Fingerman IM, LeRoy G, et al. (2009) One-pot shotgun quantitative mass spectrometry characterization of histones. J Proteome Res 8: 5367–5374.
47. Margueron R, Li G, Sarma K, Blais A, Zavadil J, et al. (2008) EZH1 and EZH2 maintain repressive chromatin through different mechanisms. Mol Cell 32: 503–518.
48. Frye SV (2010) The art of the chemical probe. Nat Chem Biol 6: 159–161.
